# Cohesin sumoylation is required for repression of subtelomeric gene expression in *Saccharomyces cerevisiae*

**DOI:** 10.1242/jcs.265023

**Published:** 2026-06-26

**Authors:** Deepash Kothiwal, Shalu Joseph, Shikha Laloraya

**Affiliations:** Department of Biochemistry, Indian Institute of Science, Bangalore 560012, India

**Keywords:** Cohesin, Silencing, Sumoylation, Budding yeast chromosome, *Saccharomyces cerevisiae*, Subtelomeric silencing

## Abstract

Cohesin, first described for its role in sister chromatid cohesion, impacts several chromosomal processes, some of which (chromosome segregation, replication and repair) are regulated by its post-translational modifications. Previously, we reported a silent information regulator (SIR) complex-independent role for cohesin in subtelomeric gene silencing. Here, we investigated the requirement of cohesin sumoylation in subtelomeric repression. We created sumoylation-deficient cohesin by fusing the catalytic domain of a SUMO protease, *ULP1*, to the C-terminus of the cohesin subunit Mcd1 (also known as Scc1) to create Mcd1-UD. We show that cohesin sumoylation is required for repression of sub-telomeric genes. Consistent with a SIR-independent role of cohesin in telomere silencing, SIR proteins remained bound to a de-repressed subtelomeric gene in *MCD1-UD-*expressing cells, and subtelomeric gene expression further increased upon *SIR2* deletion. Telomere cohesion was unaltered in this mutant, suggesting that telomere cohesion and regulation of sub-telomere gene silencing are separable functions of cohesin. Telomere tethering to the nuclear envelope and telomere compaction are defective in *MCD1-UD* cells, indicating that sumoylation contributes to the role of cohesin in subtelomeric chromosome organization. Our results establish the relevance of cohesin sumoylation in repression of subtelomeric gene expression and organization of subtelomeric chromatin.

## Teaser

**Summary:** A sumoylation defect in budding yeast cohesin results in de-repression of subtelomeric silencing, causing enhanced expression of genes near chromosome ends accompanied by impaired organization of chromosome ends.

## INTRODUCTION

Chromatin is a dynamic structure with varying organization along the chromatin fibre. Some regions are tightly compacted into transcriptionally inactive heterochromatin domains while others form less-compact, and therefore more-accessible, transcriptionally active euchromatin domains. This provides a mechanism through which gene expression can be regulated at the level of chromatin organization. In *Saccharomyces cerevisiae*, heterochromatin is found at the silent mating loci (*HML*α and *HMRa*, or the *HM* loci), telomeres and the rDNA. Heterochromatin contains hypoacetylated nucleosomes that are bound by the silent information regulator (SIR) complex, which consists of Sir2, Sir3 and Sir4. Sir2, a histone deacetylase, is the founding member of a conserved family of NAD-dependent protein deacetylases required for silencing at all three loci (Aparicio et al., 1991; Gartenberg and Smith, 2016; Kueng et al., 2013; Landry et al., 2000; Smith and Boeke, 1997). Sir3 and Sir4 are histone-binding proteins that bind with high affinity to deacetylated nucleosomes (Gartenberg and Smith, 2016; Kueng et al., 2013; Moazed, 2001). All SIR proteins (Sir1, Sir2, Sir3 and Sir4) are required for silencing of HM loci and telomeres, whereas a different protein complex, known as the RENT complex (that includes Sir2, Net1 and Cdc14) is essential for rDNA silencing (Straight et al., 1999).

As was first described in *Drosophila* (Hazelrigg et al., 1984), heterochromatin structures at telomeres result in transcriptional silencing of nearby genes, a phenomenon known as the telomere position effect (TPE). In *S. cerevisiae*, TPE was first described using reporter genes placed near modified telomeres either on the left arm of chromosome VII or the right arm of chromosome V (Gottschling et al., 1990). The effect was shown to be independent of gene identity and promoter sequence. In *S. cerevisiae*, silencing at telomeres is established by interaction and recruitment of Sir4 by the telomeric repeat-binding protein Rap1 and the telomere end-binding Yku complex proteins (Mishra and Shore, 1999; Strahl-Bolsinger et al., 1997). Sir4 in turn recruits Sir2 and Sir3; Sir2 deacetylates nearby histones thereby creating high affinity-binding sites for the SIR complex. This cycle was initially thought to continue to spread silencing in the subtelomeric region until it encounters a silencing barrier. Later studies questioned this continued spreading model and using *URA3* as silencing reporter, showed that not all telomeres are silent and that silencing is discontinuous across the length of the telomere and mostly restricted to positions close to the X element (Pryde and Louis, 1999). Moreover, despite strong enrichment of Sir proteins at telomeric repeats and core X elements, only subsets of subtelomeric genes are repressed in a SIR-dependent manner (∼6%) (Ellahi et al., 2015; Wyrick et al., 1999). Nonetheless, it has been shown that genes present within 20 kb from the chromosome end are expressed at a lower level compared to the rest of the genome (Ellahi et al., 2015; Wyrick et al., 1999) confirming TPE in *S. cerevisiae*, although the mechanism and proteins involved in this repression remained largely unknown.

The budding yeast cohesin complex consists of Smc1 and Smc3 along with two non Smc subunits, Mcd1 (also known as Scc1), the kleisin component of the complex, and Scc3 (also known as Irr1). Cohesin topologically embraces sister chromatids to provide cohesion (Hornig and Uhlmann, 2004; Ivanov and Nasmyth, 2005). Cohesin binds to centromeres, pericentromeric regions and specific sites along the chromosome arms (Blat and Kleckner, 1999; Glynn et al., 2004; Laloraya et al., 2000; Lengronne et al., 2004; Lindroos et al., 2006; Tanaka et al., 1999). In addition to its central role in sister chromatid cohesion, cohesin is also involved in various other aspects of chromosome biology such as condensation, DNA replication and repair, and regulation of gene expression (Guacci et al., 1997; Kothiwal et al., 2021; Kothiwal and Laloraya, 2019; Skibbens et al., 2010; Strom et al., 2004). Several developmental defects (collectively known as cohesinopathies) are associated with mutations in components of the cohesin network (Banerji et al., 2017; Deardorff et al., 2007; Liu et al., 2009; van der Lelij et al., 2010), and these defects could be a result of altered gene expression rather than defective sister chromatid cohesion (Gard et al., 2009; Horsfield et al., 2012). Inactivation of the cohesin complex in budding yeast has also been shown to affect the expression of a small number of genes (Skibbens et al., 2010), and we have previously shown that cohesin is required for repression of telomere proximal genes in a SIR independent manner (Kothiwal et al., 2021; Kothiwal and Laloraya, 2019).

Cohesin function is modulated by various posttranslational modifications including phosphorylation, acetylation and sumoylation. Cohesin is known to be sumoylated in both *S. cerevisiae* and humans (Almedawar et al., 2012; McAleenan et al., 2012; Stead et al., 2003; Wu et al., 2012). Wu et al. showed that Mcd1 sumoylation is required for sister chromatid recombination (SCR) upon DNA damage but not for mitotic cohesion in human cells (Wu et al., 2012). In budding yeast, all cohesin subunits undergo sumoylation in an Mms21-dependent manner, although Siz1 and Siz2 are also required for complete sumoylation (Almedawar et al., 2012; McAleenan et al., 2012; Wu et al., 2012). Using an intriguing approach of fusing the catalytic Ulp domain (UD) of the Ulp1 SUMO peptidase to the C-terminus of Mcd1, Almedawar et al. showed that cohesin sumoylation is required for its function in sister chromatid cohesion (Almedawar et al., 2012). At the same time McAleenan et al. also created a sumoylation-deficient version of Mcd1 by mutating 11 lysine residues to arginine and showed that Mcd1 sumoylation is required, independent of Chk1-mediated phosphorylation, for damage-induced cohesion, both at the site of a break and genome wide (McAleenan et al., 2012).

Having established a role of cohesin in subtelomeric gene silencing (Kothiwal and Laloraya, 2019), here, we explore the role of its sumoylation in repression of telomere proximal genes. Consistent with our observation with temperature-sensitive mutants of cohesin, we found that cohesin sumoylation is required for complete repression of telomere proximal genes such as *YFR057w* and *PAU3* independently of Sir proteins. Interestingly, Sir protein binding to de-repressed *YFR057w* and Sir silencing function were unaltered in coheisn sumoylation-deficient cells. Telomere cohesion was not reduced in the cohesin-sumoylation deficient cells indicating that telomere cohesion and subtelomeric gene repression are unlinked functions of cohesin. Furthermore. we show that reduction in sub-telomeric silencing is accompanied by telomere organization defects in the cohesin sumoylation defective cells.

## RESULTS

### Fusion of the Ulp domain to the kleisin subunit Mcd1 of the cohesin complex results in de-sumoylation of cohesin and sensitivity towards DNA damaging agents

All the cohesin subunits in *S. cerevisiae*, Smc1, Smc3, Mcd1 and Scc3, are reported to be sumoylated (Almedawar et al., 2012). The budding yeast genome codes for two SUMO proteases, Ulp1 and Ulp2. In order to understand the significance of sumoylation of the cohesin complex, the catalytic domain (UD) of Ulp1 was fused to the C-terminus of Mcd1 with 3HA as a linker in-between (referred to as Mcd1-UD) (Fig. S1A), using a strategy similar to that in Almedawar et al. (Almedawar et al., 2012). Because the kleisin component binds to and has access to both the SMC and non-SMC cohesin subunits, a Ulp domain attached to its C-terminus should be able to de-sumoylate the whole cohesin complex. As a control, we created a similar fusion protein (Mcd1-UD^FA,CS^), where the Ulp domain was inactivated by mutating residues (F474A, C580S) involved in binding to SUMO and the peptidase activity respectively (Fig. S1A). Mcd1-UD^FA,CS^ has an inactivating mutation (C580S) in the catalytic cysteine 580 in the Ulp domain and has the F474A mutation, known to prevent Ulp1 binding to SUMO; both these mutations inactivate the deSUMOylating property of UD rendering it non-functional. The steady-state level of Mcd1 was unaltered upon fusion with UD, compared to Mcd1–3HA or Mcd1-UD^FA,CS^ (Fig. S1B). We also tested the sumoylation status of the cohesin complex in Mcd1-UD using a strain expressing SUMO (Smt3) with a 6×His-Flag tag at the N-terminus. All the sumoylated proteins in the cell were pulled down using Ni-NTA resin and sumoylation of the test protein was checked by western blot analysis using anti-HA antibody in case of Mcd1 or anti-Myc antibody for Smc1 and Smc3 that were tagged with nine Myc moieties (Fig. S2A,B). Consistent with previous findings (Almedawar et al., 2012), fusion of the Ulp domain (UD) to Mcd1 diminished the sumoylation of all three tested subunits (Mcd1, Smc1 and Smc3) of the cohesin complex compared to what was seen in cells expressing the Mcd1-UD^FA,CS^ (hereafter referred to as *MCD1-ud*) (Fig. S2A,B), suggesting that Mcd1-UD can indeed de-sumoylate other subunits of the cohesin complex.

We also tested the growth phenotype of *MCD1-UD*, relative to *MCD1-ud* or wild-type cells by spot assay at various temperatures and in the presence of DNA-damaging agents (Fig. S3). The strain harboring the *MCD1-UD* allele showed a growth defect at 23°C but grew comparably to *MCD1-ud* and wild-type cells at 30°C and 37°C (Fig. S3, top panels). Similar to other SUMOylation-defective cohesin mutants (McAleenan et al., 2012; Wu et al., 2012), *MCD1-UD* cells were sensitive towards hydroxyurea (HU) and methyl methanesulfonate (MMS) (Fig. S3, bottom panels), suggesting a role of cohesin sumoylation in countering replication stress and DNA damage. Under all the conditions tested, *MCD1-ud* (the strain expressing *MCD1* fused to the catalytically inactive UD^FA,CS^ mutant) grew as well as the wild-type, therefore in all our further studies, *MCD1-ud* was used as the wild-type control.

### Cohesin sumoylation is required for repression of telomere proximal genes

**Fig. 1.**
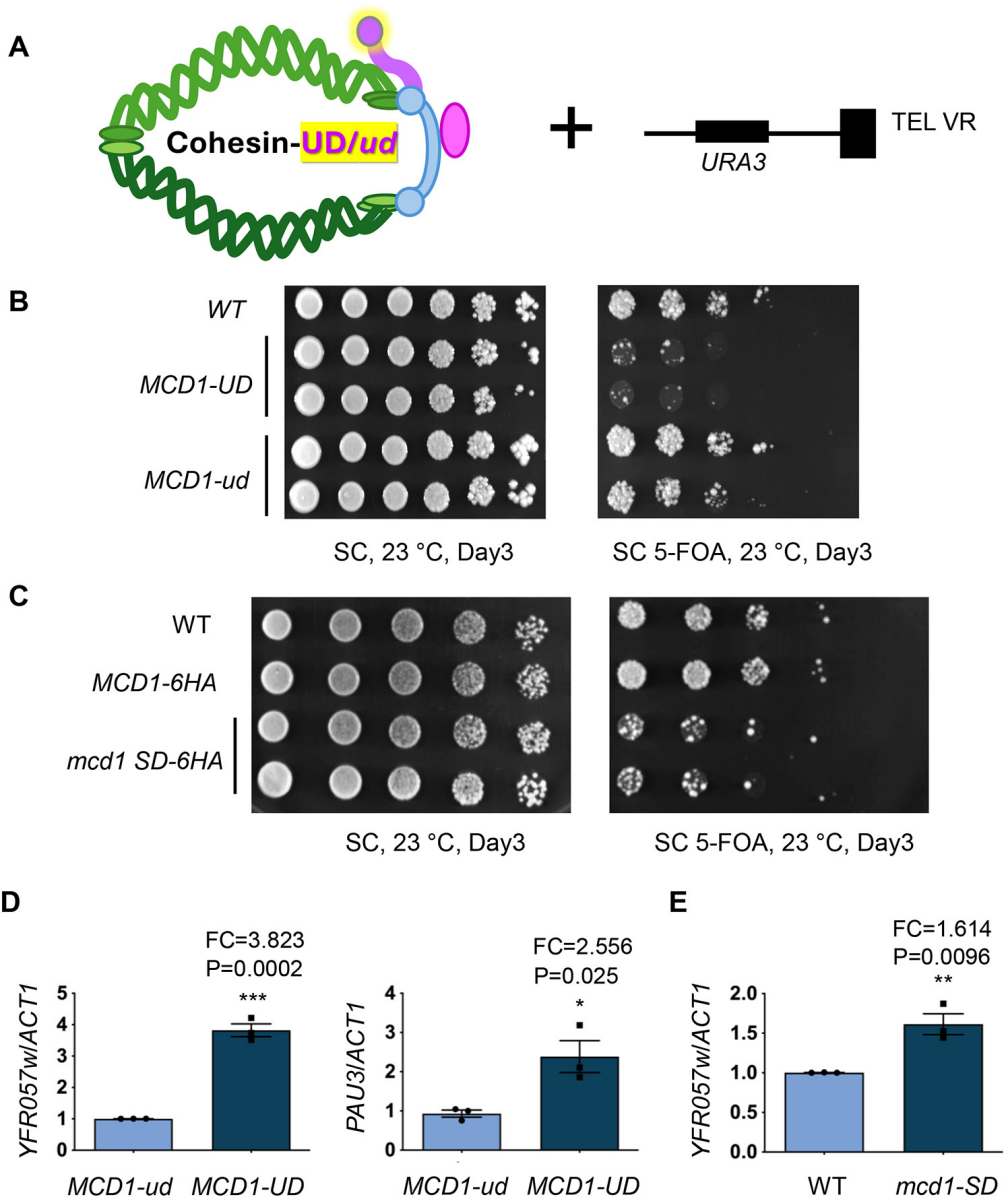
**Telomere silencing defect in cohesin sumoylation-defective cells.** (A) Schematics of the cohesin-UD fusion (left) and modified telomere (TEL) VR having a *URA3* silencing reporter (right). The UD catalytic domain is represented by the glowing purple ball attached to the Mcd1 subunit, shown in blue. (B) TPE measurement in ROY783. Overnight grown log phase cultures of wild-type (WT; SLY1807), *MCD1-UD* (SLY1820) and *MCD1-ud* (SLY1823) strains were serially diluted and spotted onto SC and SC+0.1% 5-FOA plates. Plates were incubated at 23°C for the indicated time. (C) Spot test to analyze silencing of telomere-proximal *URA3* reporter at 23°C in wild-type (WT; ROY783), *MCD1-6HA* (SLY2743) and *mcd1 SD-6HA* (SLY2745) strains. Images shown in B and C are representative of five biological replicates. (D,E) Expression levels of native telomere proximal genes in *MCD1-UD* and *mcd1-SD.* RT-qPCR analysis to compare transcript levels of the sub-telomeric genes, *YFR057w* and *PAU3* in *MCD1-ud* (SLY1823) and *MCD1-UD* (SLY1820) strains (D), and *YFR057w* in WT (SLY2743) and *mcd1-SD* (SLY2745) (E). The mean values for experiments with *n*=3 independent biological replicates are plotted on the *y*-axis. Error bars indicate s.e.m. FC, fold change. *P≤0.05; **P≤0.01; ***P≤0.001 (two-tailed unpaired *t*-test).

To test the role of cohesin sumoylation in telomere silencing, we performed a widely used telomere silencing assay using a strain having a *URA3* reporter gene inserted near a modified telomere VR (Fig. 1A) (Donze et al., 1999). *URA3* expression can be qualitatively measured in terms of growth on 5-fluoroorotic acid (5-FOA) containing plates. 5-FOA is a counter selection marker for *URA3,* therefore growth on 5-FOA containing plates is inversely proportional to *URA3* expression. *MCD1-UD* or *MCD1-ud* were introduced in a strain having the *URA3* silencing reporter near telomere VR (Fig. 1A) and silencing was assayed by assessing growth in presence of 5-FOA. We observed that *MCD1-UD* was highly sensitive to 5-FOA compared to *MCD1-ud* or wild-type cells, suggesting an increase in *URA3* expression and loss of silencing at telomere VR (Fig. 1B).

There is a possibility that *MCD1-UD* might de-sumoylate nearby proteins that are not part of the cohesin complex and that the de-sumoylation of such non-targeted proteins results in loss of silencing at telomeres rather than cohesin de-sumoylation. To address this possibility, we created a sumoylation-deficient *MCD1* allele (*mcd1-SD*), by mutating 11 lysine residues to arginine (Fig. S1C). This allele has been shown to be defective in Mcd1 sumoylation (McAleenan et al., 2012). Using the same *URA3* reporter-based telomere silencing assay, we found that, similar to *MCD1-UD*, *mcd1-SD* cells are also sensitive to 5-FOA (Fig. 1C), although to a lesser extent, probably because this mutant is defective only in Mcd1 sumoylation and not sumoylation of other cohesin subunits. These results confirm that impairment of cohesin sumoylation can indeed result in decrease of telomere silencing.

To test whether cohesin sumoylation is required for silencing of natural subtelomeric genes, we chose to examine the transcript levels of the native subtelomeric genes *YFR057w* and *PAU3*, whose silencing is known to be cohesin dependent (Kothiwal and Laloraya, 2019), by reverse transcriptase real-time quantitative PCR (RT-qPCR). We observed that *YFR057w* and *PAU3* were significantly de-repressed in *MCD1-UD* as compared to *MCD1-ud* (Fig. 1D), further confirming that cohesin sumoylation is indeed required for complete silencing of subtelomeric genes. In addition, *mcd1-SD* cells also showed de-repression of *YFR057w* (Fig. 1E).

### Binding of silencing regulators to chromatin is unaltered in cohesin sumoylation deficient cells

Loss of silencing observed in non-sumoylatable cohesin mutants could arise from changes in the expression level of proteins involved in establishment and spreading of silencing. Silencing at telomeres is thought to be dependent on the SIR complex proteins Sir2, Sir3 and Sir4. To compare the steady-state levels of these silencing proteins, we created Sir3–9Myc and Sir4–9Myc by epitope tagging them at the C-terminus, whereas anti-Sir2 antibody was used to detect native Sir2 protein. We did not find any reduction in the steady-state expression levels of these proteins in cohesin sumoylation-deficient cells by western blot analysis using whole-cell lysates (Fig. S4). Furthermore, we did not observe any change in the steady-state levels of Rap1 (Fig. S4), a factor involved in recruitment of Sir proteins to the telomeres.

**Fig. 2.**
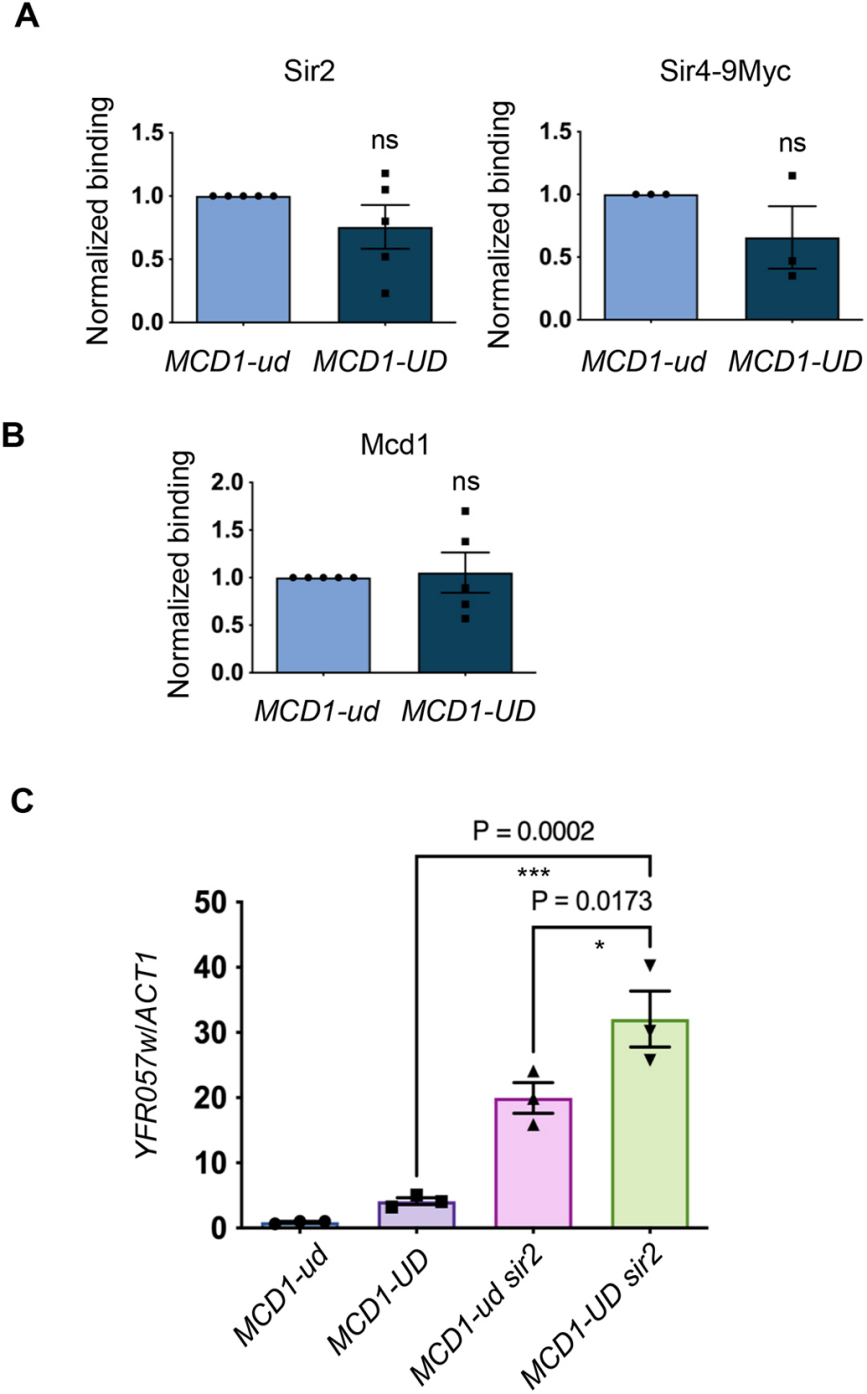
**Cohesin sumoylation mediated repression of subtelomeric genes is independent of Sir proteins.** (A) Binding of Sir proteins at *YFR057w* by ChIP-qPCR. Histograms for Sir2 and Sir4-9Myc binding are shown. In the ChIP assay, the fold enrichment levels were calculated relative to *PHO5.* The mean values for *n*≥3 experiments are plotted on the *y*-axis, *n*=5 for Sir2 and *n*=3 for Sir4–9Myc (independent biological replicates). Error bars indicate s.e.m. For simplicity wild-type values were normalized to 1. (B) ChIP-qPCR to analyze Mcd1 binding at 500 bp from telomere VIR in *MCD1-ud* (SLY1823) and *MCD1-UD* (SLY1820) cells; *n*=5 biological replicates. ns, not significant (two-tailed unpaired *t*-test). (C) RT-qPCR analysis to measure expression level of *YFR057w* in *MCD1-ud* (SLY1823), *MCD1-UD* (SLY1820) compared to *MCD1-ud sir2* (SLY2911) and *MCD1-UD sir2* (SLY2912). Expression levels are measured relative to *ACT1.* The mean values for *n*=3 experiments from three independent biological replicates are plotted on the *y*-axis. Error bars indicate s.e.m. *P≤0.05 (P=0.0173); ***P≤0.001 (P=0.0002) (two-way ANOVA with multiple comparison).

Given that there were no changes in the steady-state level of Sir proteins, we examined the binding of Sir2 and Sir4 at *YFR057w*, a gene present near telomere VIR whose expression is increased in the cohesin sumoylation-defective cells, by chromatin immunoprecipitation (ChIP). Interestingly, we did not observe significant change in the binding of Sir proteins at this site in the sumoylation-deficient mutant compared to *MCD1-ud* (Fig. 2A). Previously, we have shown that though Sir binding to telomeres was not altered in cohesin temperature-sensitive mutants, cohesin binding was decreased (Kothiwal and Laloraya, 2019). We tested for such a possibility by analyzing Mcd1 binding as proxy for the cohesin complex and observed no change in cohesin binding (Fig. 2B) suggesting that sumoylation affects some other property of the complex which is required for transcriptional repression near telomeres.

### Cohesin sumoylation contributes to subtelomeric gene silencing independent of Sir proteins

Since we did not observe any difference in the association of Sir proteins with the de-repressed subtelomeric gene, *YFR057w*, we wondered whether the role of cohesin sumoylation in subtelomere silencing could be Sir independent. To test this possibility, we compared the transcript levels of *YFR057w* in *MCD1-UD sir2* double mutant with *MCD1-UD*, *MCD1-ud* (WT) and *MCD1-ud sir2*. We found that *YFR057w* transcripts were more abundant in double mutants relative to either of the single mutant or WT cells (Fig. 2C), suggesting that cohesin sumoylation contributes independently of Sir proteins for complete silencing of the telomere proximal gene.

### Sir proteins are competent to establish silencing in cohesin sumoylation-defective cells

**Fig. 3.**
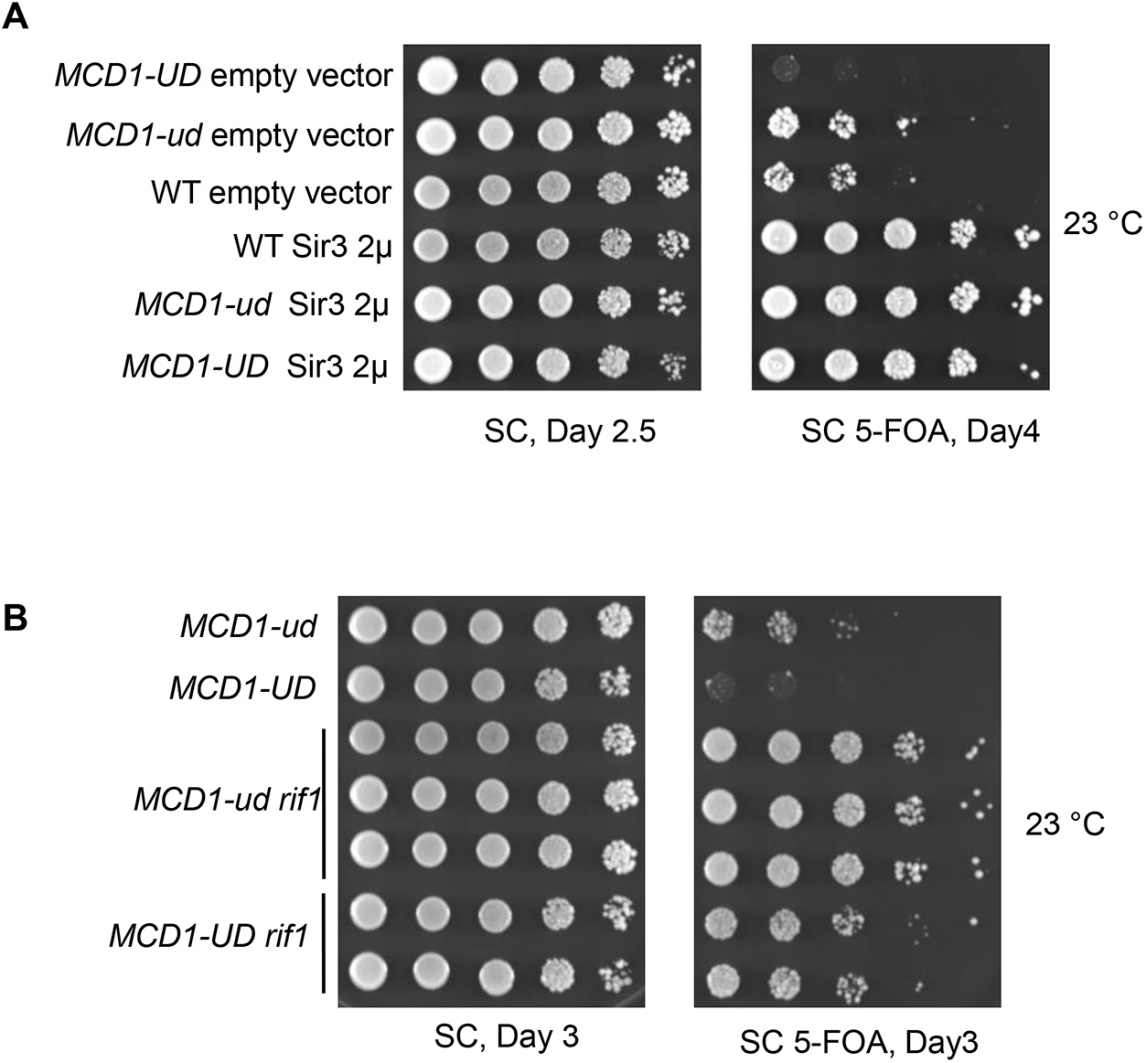
**Sir proteins are silencing competent in *MCD1-UD*.** Genetic analysis of Sir protein function in *MCD1-UD,* using a strain bearing telomere proximal *URA3.* (A) Sir3 overexpression suppresses the silencing defect in *MCD1-UD*. TPE determination upon Sir3 overexpression from a 2 μ plasmid (pRO146). Overnight grown log phase cultures were serially diluted and spotted onto SC-Trp and SC-Trp+0.1% 5-FOA plates at 23°C in wild-type (WT; ROY783), *MCD1-ud* (SLY1823) and *MCD1-UD* (SLY1820) strains. Representative images from two independent experiments are shown. (B) Suppression of TPE defect by *RIF1* deletion. Dilution spotting for TPE analysis on SC and SC+0.1% 5-FOA plates at 23°C in *MCD1-ud* (SLY1823), *MCD1-UD* (SLY1820), *MCD1-ud rif1* (SLY2526) and *MCD1-UD rif1* (SLY2528). Images shown are representative of three independent repeats.

As the binding of Sir proteins to the telomeric heterochromatin was not reduced in the mutant, we wondered whether these silencing regulators were competent to establish silencing in this mutant. To test this, we carried out genetic interaction analysis with *RIF1* and *SIR3*. Sir3 overexpression can extend the zone of silencing at telomeres, as it can bind beyond the SIR complex-binding sites independently of other Sir proteins (Hecht et al., 1996; Renauld et al., 1993). Sir3 overexpression also leads to gain of silencing at telomeres (Hecht et al., 1996; Renauld et al., 1993). Consistent with this, we observed increased silencing of the *URA3* reporter upon overexpression of Sir3 (Fig. 3A). Interestingly, Sir3 overexpression in *MCD1-UD* led to complete repression of *URA3* to a level equivalent to that in the wild-type or *MCD1-ud* (Fig. 3A), suggesting that Sir proteins are competent to establish silencing and their overexpression suppresses the silencing defect of the sumoylation defective cohesin mutant, at least at the modified telomere VR.

Rif1 is a Rap1 interaction factor (Hardy et al., 1992) that competes with Sir4 for binding at the telomeres. In a *rif1* deletion mutant, binding of Sir proteins to the telomeres increases, resulting in gain of silencing (Kyrion et al., 1993; Wotton and Shore, 1997). Deletion of *RIF1* resulted in significant enhancement of silencing of the *URA3* reporter in the *MCD1-UD rif1* double mutant relative to *MCD1-UD* (Fig. 3B). Together, these observations indicate that Sir proteins retain their silencing function in *MCD1-UD* and the transcriptional de-repression in this mutant is not due to a defect in the silencing function of Sir proteins.

### Telomere length maintenance in cohesin sumoylation-defective cells

Telomeres are repetitive regions at the ends of the chromosomes, and their length must be maintained for genome stability. The cell uses various mechanisms to maintain telomere length homeostasis. Telomere length also has a causal relationship with silencing as reduction in telomere length leads to decrease in Rap1 binding sites and hence reduced SIR binding and de-repression of subtelomeric genes. To test whether de-repression of subtelomeric genes could be a result of decrease in telomere length, we examined telomere length in *MCD1-UD* using a Y′ element specific probe (Fig. S5A). Interestingly, rather than shortening, we observed a slight increase in telomere length in *MCD1-UD* compared to wild-type cells and to cells expressing Mcd1 fused to the UD inactivation mutant (*MCD1-ud*) (Fig. S5B), suggesting that decrease in expression of telomere proximal genes is not due to telomere shortening in the cohesin sumoylation-defective cells. It also indicates that sumoylation of the cohesin complex might be important for telomere length maintenance.

### Analysis of sister chromatid cohesion in the cohesin sumoylation-defective cells

**Fig. 4.**
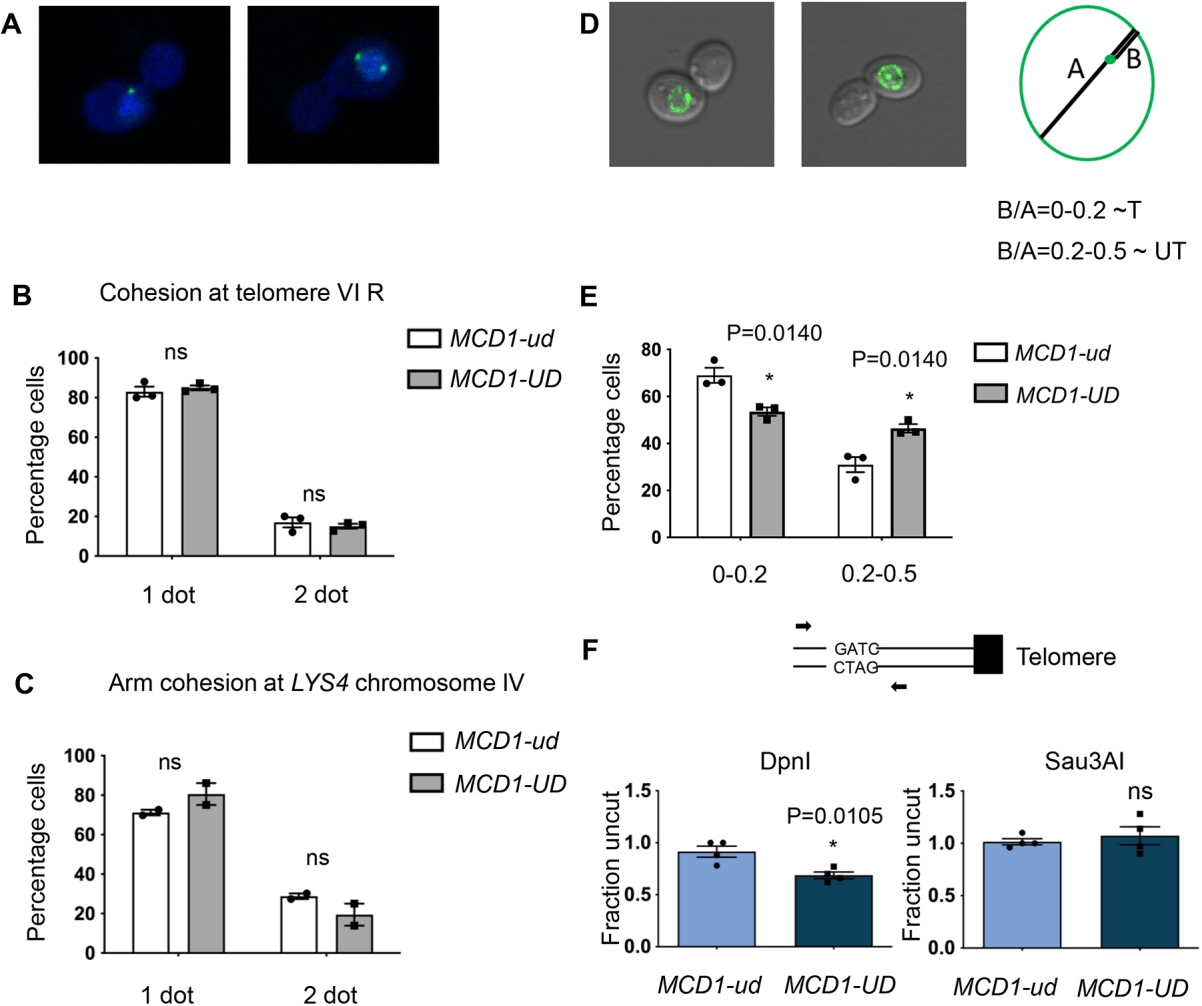
**Telomere organization in cohesin sumoylation defective cells.** (A) Analysis of sister chromatid cohesion in *MCD1-UD* cells at 23°C. A merged image of DAPI and GFP showing cohered (left) and non-cohered (right) configurations. (B) Analysis of cohesion at a telomere proximal site in *MCD1-ud* (SLY2446) and *MCD1-UD* (SLY2448) cells carrying a CFP chromosomal tag near telomere VIR. Three independent biological replicates were used in each case. Error bars indicate s.e.m.  ns, not significant (two-tailed unpaired *t*-test). (C) Analysis of sister chromatid cohesion at *LYS4* in *MCD1-ud* (SLY2549) and *MCD1-UD* (SLY2551) cells carrying a chromosomal GFP -tag on the right arm of chromosome IV. The percentage of cells showing cohesion (one dot) or loss of cohesion (two dots) were scored for wild-type and *MCD1-UD* cells, two independent biological replicates were used in each case. The mean values are plotted on the *y*-axis. Error bars indicate s.e.m. ns, not significant (two-tailed unpaired *t*-test). (D) Tethering of GFP-tagged TEL-VR to the nuclear envelope was measured in nocodazole-arrested *MCD1-ud* (SLY2557) and *MCD1-UD* (SLY2559) strains expressing *NUP49-GFP.* Representative images showing tethered (left) and untethered (right) configurations. A B/A value of 0–0.2 was considered as tethered whereas 0.2–0.5 was considered as untethered (where A is the nuclear diameter and B is distance of the telomere dot from the nuclear envelope). (E) The graph showing percentage of cells having tethered versus untethered telomeres in WT (*MCD1-ud*) versus *MCD1-UD*. Three independent biological replicates were scored. Error bars indicate s.e.m. *P≤0.05 (two-tailed unpaired *t*-test). (F) Assessment of chromatin accessibility at *YFR057w* by the Dam methylase accessibility assay in *MCD1-ud* (SLY2996) and *MCD1-UD* (SLY2998) cells by digestion with DpnI (left) or Sau3AI (right) followed by qPCR, *n*=4 independent biological replicates. The schematic on top depicts locations of primers (arrows) near TEL-VIR relative to the cutting site. The mean values for *n*≥3 experiments are plotted on the *y*-axis. Error bars indicate s.e.m. *P≤0.05; ns, not significant (two-tailed unpaired *t*-test).

Cohesin has an established role in sister chromatid cohesion (Guacci et al., 1997; Michaelis et al., 1997), in which it tethers two sister DNA molecules to bring about their cohesion. We examined sister chromatid cohesion at both a chromosome arm and a telomere in *MCD1-UD* cells at 23°C. Cohesion was assayed using strains expressing GFP or CFP tagged Lac-I, bound to tandem *lacO* repeats integrated at *LYS4* on right arm of chromosome IV (Neurohr et al., 2011) or near telomere VIR (Bystricky et al., 2005) respectively. Cohesion was measured in nocodazole treated G2/M arrested cells. When chromatids are cohesed, only a single fluorescent dot is visible whereas loss of cohesion leads to appearance of two dots, each belonging to a sister chromatid (Fig. 4A). Using this assay we did not observe any significant change in cohesion, either at the telomere or at the arm site (Fig. 4B,C, respectively), indicating that cohesin sumoylation is not required for sister chromatid cohesion at these tested sites. Interestingly, it also suggests that requirement of cohesin for transcriptional repression of subtelomeric genes is independent of its role in telomere cohesion.

### Cohesin sumoylation is required for efficient telomere tethering to the nuclear envelope

The peripheral nuclear space adjacent to the inner nuclear membrane has been described as a sub-compartment of the nucleus that favors silencing of genes localized in this region (Feuerbach et al., 2002; Hediger and Gasser, 2002). Telomeres are tethered to the nuclear envelope, and it has also been proposed that that tethering plays an important role in silencing (Feuerbach et al., 2002; Hediger and Gasser, 2002). Moreover, we have previously shown that cohesin is required for efficient telomere tethering to the nuclear envelope (Kothiwal and Laloraya, 2019). To test whether sumoylation is required for this function of cohesin, we examined the effect of cohesin de-sumoylation on telomere tethering to the nuclear envelope. We used a strain expressing GFP tagged Lac-I bound to tandem repeats of *lacO* integrated near telomere VR marking the telomere (Bystricky et al., 2005) in which the nuclear envelope was visualized by GFP tagging of Nup49 (a nuclear envelope protein). The percentage of nuclei having tethered or untethered configurations of the telomere was enumerated in wild-type and *MCD1-UD* cells grown at 23°C and arrested in G2/M with nocodazole. Tethering was calculated by measuring the ratio of the distance of the telomere dot from the nuclear envelope (B) to the diameter of the nucleus (A). A B/A value ranging from 0–0.2 was considered as tethered whereas a B/A value between 0.2 and 0.5 was considered as untethered (Fig. 4D). We found a modest but statistically significant reduction in tethering of telomere VR to the nuclear envelope and a concomitant increase in untethering in *MCD1-UD* cells (from 31% in WT to 46.45% in the mutant) (Fig. 4E). This finding indicates that cohesin sumoylation contributes to nuclear membrane tethering of the telomere in G2/M cells.

### Cohesin sumoylation is required for compaction of the telomere proximal region

Previous studies demonstrated that telomeres have a special compact organization, which is refractory to DNA-modifying enzymes (Gottschling, 1992). We have shown that both Sir2 and cohesin are independently required for the formation of this specialized structure (Kothiwal and Laloraya, 2019). To understand the regulation of this function of cohesin we compared chromatin compaction in *MCD1-ud* and *MCD1-UD* cells by quantifying Dam methylase (an enzyme that methylates adenine within a GATC sequence) accessibility of sub-telomeric chromatin near Tel-VIR (Fig. 4F, top graphic) (Gottschling, 1992). Sub-telomeric chromatin in the sumoylation-defective mutant appeared to be more accessible as it was more susceptible to cleavage by DpnI, indicating a relatively open organization (Fig. 4F, left panel). The same region was equally sensitive to Sau3A1, a methylation-insensitive restriction endonuclease (Fig. 4F, right panel). These findings indicate that subtelomeric chromatin in *MCD1-UD* assumes a more open conformation relative to that in *MCD1-ud* cells. Interestingly, unlike *mcd1-1*, a non-subtelomeric region (*YFL015C*) remained equally accessible irrespective of the sumoylation status of cohesin (Fig. S6).

## DISCUSSION

Cohesin is known to undergo various posttranslational modifications, such as acetylation, phosphorylation and sumoylation, and these modifications modulate its activity (Rolef Ben-Shahar et al., 2008; Unal et al., 2008; Zhang et al., 2008). Cohesin plays many other important roles beyond its central role in sister chromatid cohesion. Cohesin also has a well-established role in regulation of gene expression, and many developmental defects associated with mutations in the cohesin network proteins are believed to be a result of altered gene expression. Surprisingly, no study has directly evaluated the role of posttranslational modifications of cohesin in the regulation of gene expression.

Here, we have investigated the role of cohesin sumoylation in subtelomeric gene silencing. Using *MCD1-UD*, we show that cohesin sumoylation is required for silencing of the telomere silencing reporter *URA3* and subtelomeric genes *YFR057w* and *PAU3* (Fig. 1), although the effect of cohesin de-sumoylation on silencing is not as strong as was observed with temperature-sensitive point mutants of the cohesin complex (Kothiwal and Laloraya, 2019). In contrast to Almedawar et al., who found that downregulation of cohesin sumoylation is lethal (as *SCC1-UD* made by them does not complement the thermosensitive phenotype of *scc1-73* cells), we found that the *MCD1-UD* fusion construct made by us supports viability at 30°C and 37°C (although cells harboring it are slightly slow growing at 23°C) and sister chromatid cohesion, at least at the tested loci near the telomere (*TEL VIR*) and an arm site (*LYS4* on CHR IV right arm). These differences could arise due to different strain backgrounds used in these studies. Moreover, Almedawar et al. found a decrease in sumoylation of Scc3 and Pds5 upon overexpressing Scc1-UD, but the desumoylation of these subunits was not tested when Scc1-UD was expressed from its own promoter. We have also not tested the desumoylation status of these two proteins with our construct Mcd1-UD, which is also expressed from its endogenous promoter. It is possible that sumoylation of cohesin subunits might not have reduced as much under our conditions, which could be important for survival. Nevertheless, the viability of Mcd1-UD expressing cells allowed us to assay telomeric silencing using the *URA3* reporter as well as a subtelomeric gene *YFR057w* and *PAU3* in this system uncomplicated by a layer of heat-shock response induction that would be unavoidable if we were to use the temperature-sensitive *scc1-73* background as in the other study.

We found that SIR protein binding is retained at *YFR057w* in the sumoylation-defective cohesin mutant and hence cohesin sumoylation might act in parallel to or partially independently of Sir proteins for repression of this subtelomeric gene (Fig. 2B,C). These observations are consistent with our earlier finding using temperature-sensitive mutants of the cohesin complex in which it was found that the role of cohesin in subtelomeric silencing is SIR independent (Kothiwal and Laloraya, 2019). We also show that cohesin sumoylation is required for telomere organization as cohesin sumoylation-deficient cells have slightly longer telomeres (Fig. S5) and are defective in telomere anchoring to the nuclear envelope and telomere compaction (Fig. 4D–F).

To understand the role of sister chromatid cohesion in transcriptional silencing of telomere proximal regions, we analyzed cohesion at a telomere and an arm site. Interestingly, we observed that cohesion was intact at both the loci. It has been difficult to separate sister chromatid cohesion from other functions of cohesin as most of the available mutants are defective in cohesion as well. Our observation suggests that telomere cohesion and subtelomeric transcriptional silencing are separable functions of cohesin. Moreover, no loss of cohesion or change in cohesin binding was observed in cohesin sumoylation-defective cells, suggesting that cohesin uses different mechanisms for chromatin compaction and sister chromatid cohesion, at least at telomeres.

Based on these observations we propose that sumoylation is required for the function of cohesin in repression of expression of telomere proximal genes acting in parallel with the SIR-mediated silencing pathway, possibly via its role in telomere organization.

## MATERIALS AND METHODS

### Media and reagents

Cells were grown in YPD medium (1% yeast extract, 2% peptone and 2% dextrose; 2% agar was added for plates) or synthetic complete (SC) medium (0.67% yeast nitrogen base without amino acids, 2% dextrose and complete supplement/dropout mix as required; 2% agar was added for plates) as indicated. We purchased G418 from Sigma, hygromycin from US Biological, nourseothricin from Jena bioscience; 12CA5 anti-HA, 9E10 anti-Myc and anti-GFP (11814460001) antibodies from Roche Applied Science, sc-25753 anti-SIR2 (y-80) (polyclonal rabbit antibody) from Santa Cruz Biotechnology and HRP-conjugated goat anti-mouse IgG (1140680011730, HPO6) and goat anti-rabbit IgG (1140380011730, HPO3) secondary antibodies from Genei Laboratories Private Limited.

### Yeast strains and plasmids

The yeast strains used or generated in this study are listed in Table S1. Oligonucleotides used for construction of strains and plasmids were synthesized by Sigma-Aldrich; sequences of these oligonucleotides are provided in Table S2. The 2 μ SIR3 overexpression plasmid pRO146 was a gift from R. Kamakaka (Donze et al., 1999).

#### Integration of *MCD1-UD* or *MCD1-ud* at the *MCD1* genomic locus

To integrate *MCD1-UD*, the integration cassette (*MCD1-UD*:*LEU2*) was created in three steps. In the first step, *MCD1* downstream sequence (sequence after the stop codon) was cloned into HincII-digested pUC19 to generate pDK16. In the second step, a BglII fragment of pSL617 (*sir2*Δ*::LEU2*) was cloned into the BamHI site of pDK16 to give rise pDK17. In the final step, *MCD1-UD* was PCR amplified from pDK6 using SLO505 and SLO508 and cloned into the SmaI site of pDK17 and named as pDK18. To integrate *MCD1-UD*, a HindIII-digested fragment of pDK18 was transformed into different yeast strains. Integrations were confirmed by colony PCR using the primers LEU L2B and SLO613; a fragment of 610 bp is formed upon correct integration.

The *MCD1-ud* integration cassette was generated in a similar way, except that *MCD1-ud* was created by site-directed mutagenesis using pDK6 as a template and cloned into the SmaI site of pDK17, the integration and confirmation strategies were also the same.

#### Integration of *mcd1 SD* or *MCD1*-6HA at the *MCD1* genomic locus

To integrate *mcd1 SD* (*mcd1 11KR*), the integration cassette (*mcd1 SD-6HA:hphNT1 kanMX6*, pDK28) was created in two steps. In the first step *mcd1 SD-6HA:hphNT1* was created by site-directed mutagenesis using *MCD1-6HA:hphNT1* as a template and cloned into the SmaI site of pUC19 to generate pDK25. The lysine residues (K165, K252, K290, K345, K391, K392, K394, K460, K500, K509 and K521) were mutated to arginine to generate *mcd1-SD*. In the second step, the *kanMX6*-containing fragment of pFA6a-kanMX6 was cloned into the BstZI7I site of pDK25 to generate pDK28. To integrate *mcd1 SD*, a PsiI-digested fragment of pDK28 was transformed into yeast strain ROY783. Integrations were confirmed by colony PCR using oligonucleotides SLO791 and SLO613, which yields a fragment of ∼450 bp upon correct integration.

The *MCD1-6HA:hphNT1 kanMX6* (pDK33) cassette was created in a similar way. The integration and confirmation strategies were also the same.

#### Creation of the *RIF1* deletion strain

The *RIF1* deletion cassette was amplified from SLY859 (*rif1*Δ*::KanMX4*) using SLO882 and SLO883. Yeast cells were transformed with the ethanol-precipitated PCR product, transformants were selected on 200 μg/ml G418 plates and deletion was confirmed by colony PCR using SLO441 and SLO884, which produces a fragment of ∼400 bp.

#### Construction of the HF-Smt3-expressing strains

The plasmid bearing *HF-SMT3* (pYRTAG310) was transformed into W303-1a and the genomic copy of *SMT3* was knocked out by PCR-mediated one-step gene disruption (Janke et al., 2004) using primers SLO816/SLO817 and pYM16 as the template. Genomic disruption of *SMT3* was confirmed by colony PCR of hygromycin-resistant transformants (hygromycin-B used at 300 μg/ml) using primers SLO818/SLO534, which produce a 620 bp fragment upon correct gene disruption.

#### Construction of the *SIR2* deletion strains

*SIR2* was deleted using PCR-mediated one-step gene disruption (Janke et al., 2004). To delete *SIR2*, the primer pair SLO679/SLO680 was used to amplify the deletion cassette using pYM22 as the template. Genomic disruption of *SIR2* was confirmed by PCR using the oligonucleotide pair SLO791/SLO024, which produces a fragment of ∼600 bp upon *SIR2* deletion.

#### Creation of *SIR3*-, *SIR4*- and *RAP1*-tagged strains

All the proteins were epitope tagged at the C-terminus using PCR mediated one-step tagging approach (Janke et al., 2004): *SIR3* was tagged with 9Myc using the primer pair SLO876/877 and pYM20 as template; for *SIR4*, 9Myc tagging was performed using pYM20 as the template for PCR with the primer pair SLO871/872 to amplify the myc-tagged cassette; for *RAP1*, GFP tagging of *RAP1* was done using pYM27 as the template and using primers SLO907 and SLO908 to amplify the tagging cassette.

### Spot assay

To assay growth and drug sensitivity (as in Fig. S3), yeast cells were grown in YPD medium until mid-log phase (OD_600_ of 0.8–1.0). Cultures were serially diluted ten-fold starting from 10^−1^ and spotted on YPD plates with and without drugs (e.g. HU and MMS). YPD plates were incubated at various temperatures (23°C, 30°C and 37°C). MMS (0.01%) and HU (100 mM) plates were maintained at 23⁰C. Plates were incubated for 3–5 days and images were captured at the mentioned intervals/time points.

### Preparation of whole-cell extracts and immunoblot analysis

Cells were grown to mid-log phase (OD_600_ ∼0.8) and protein extracts were prepared by lysing the cells in trichloroacetic acid (TCA) using glass beads. Samples were resolved by SDS-PAGE. The resolved proteins were transferred onto a nitrocellulose membrane using the Bio-Rad semi dry apparatus (39 mM glycine, 48 mM Tris-HCl and 15% methanol). Proteins were detected using indicated primary antibodies and HRP-conjugated secondary antibody. Primary antibodies were used at a dilution of 1:1000 and secondary antibodies at a dilution of 1:5000. Blots were developed using Perkin-Elmer chemiluminescence reagent. Uncropped images of western and Southern blots are shown in Figs S7–S10.

### Pull-down assay to detect sumoylation

Pull-down was performed as described previously (McAleenan et al., 2012), with minor modifications. To detect His-FLAG tagged SUMO conjugates from yeast cells, 600 ml cultures were harvested and disrupted by glass beads in 1 ml Buffer A (8 M Urea, 100 mM NaH_2_PO_4_, 10 mM Tris-HCl, 0.05% Tween 20, pH 8), and extracts were clarified by centrifugation at 13,000 ***g*** for 10 min at 4°C. Protein concentration was determined using the Bradford Assay, and ∼12–15 mg of protein was added to 50 µl of 50:50 slurry of Ni-NTA beads (prewashed in Buffer A). Imidazole was added to a final concentration of 20 mM, to reduce non-specific binding. Proteins were bound to the beads for 3 h at 4°C on a rotating platform. Protein bound beads were washed twice with Buffer A containing 2 mM imidazole, followed by two washes in Buffer B (8 M Urea, 100 mM NaH_2_PO_4_, 10 mM Tris-HCl, 0.05% Tween 20, pH 6.3). Bound proteins were eluted off the beads using 30 μl of 2× Laemmli buffer supplemented with 4% β-mercaptoethanol and 200 mM EDTA and the samples were subjected to SDS-PAGE followed by western blotting.

### Telomere silencing assay

Overnight grown log phase cultures were normalized to 1 OD_600_ and serially diluted to OD_600_ 0.5, 0.1, 0.01 and 0.001, and spotted on SC and SC 5-FOA (1 mg/ml or 0.1%) plates. Plates were incubated at 23°C and images were captured at intervals including the indicated time points.

### RT-qPCR

Total RNA was isolated from exponentially growing yeast cells using Qiagen RNeasy minikit. cDNA was prepared from 2 μg of DNase treated RNA, and qPCR was carried out on a Bio-rad IQ5 real time PCR machine using Bio-rad iTaq universal SYBR green supermix. The primers used for qPCR are listed in Table S3. The expression level of test genes in all cases was normalized with respect to *ACT1.* The mean values for *n*≥3 experiments are plotted on the *y*-axis. Error bars represent the s.e.m. An unpaired two-tailed Student's *t*-test was used to evaluate the statistical significance of the observed differences in expression (*P≤0.05, **P≤0.01 and ***P≤0.001).

### ChIP-qPCR

ChIP was performed as described previously (Laloraya et al., 2000) using asynchronous cultures. qPCR of ChIP samples was done in a Bio-rad IQ5 real-time PCR machine using Bio-rad iTaq universal SYBR Green supermix. Calculations were done using ΔΔCt methods where Ct values at the test locus were normalized with respect to total and a control locus (as indicated in figure legends) (Kothiwal and Laloraya, 2019). The primers used for qPCR are listed in Table S3. The fold enrichment is relative to the *PHO5* in all ChIP-qPCR experiments. The mean values for *n*≥3 experiments are plotted on the *y*-axis. Error bars represent the s.e.m. For simplicity, the wild type values were normalized to 1.

### Southern blot

Genomic DNA for Southern blot analysis was isolated from exponentially growing yeast cell cultures by glass bead lysis (Adams et al., 1997). The DNA pellet obtained was resuspended in 40–50 μl of sterile water. 6.0–8.0 μg of the Xho1 digested genomic DNA was electrophoresed on a 1% agarose gel in 1× TAE (40 mM Tris 20 mM acetate, 1 mM EDTA) buffer and then transferred onto Hybond-N nylon membranes (GE Healthcare) overnight at room temperature using 10× SSC (1.5 M NaCl, 0.15 M Na-citrate) as the transfer buffer. The blot was UV-crosslinked using an Amersham UVC 500 crosslinker for 5 min. To examine telomere lengths, the membrane was probed with a [^32^P] radiolabeled probe specific for the Y′ element, obtained from pRR48. The radiolabeled probe was generated by random priming using α-[^32^P]dATP in a reaction containing template DNA (25–50 ng), 0.5 mM dNTPs, Klenow polymerase buffer, random primer and Klenow polymerase. Signal intensities were obtained by detecting photo-stimulated luminescence counts in a GE Typhoon FLA9000 phosphorimager.

### Microscopy

For the cohesion assay, exponentially growing cultures were supplemented with 1% DMSO for 30 min and nocodazole (Sigma) was added to a final concentration of 15 µg/ml. Metaphase-arrested cells carrying GFP or CFP chromosome tags were analyzed by confocal microscopy, where 3 µl (1 mg/ml) DAPI was added for DNA staining 20 min prior to harvesting. Cells were immediately fixed with 4% paraformaldehyde, and imaging was performed using fixed cells. Experiment was repeated three times and each time ∼100 cells were counted for each wild-type and mutant.

Similarly for telomere tethering, exponentially growing cultures were supplemented with 1% DMSO, for 30 min and nocodazole added to a final concentration of 15 μg/ml. Metaphase-arrested cells expressing *NUP49-GFP* and having a GFP chromosome tag were analyzed by confocal microscopy. Cells were immediately fixed with 4% paraformaldehyde and imaging was performed using fixed cells. The experiment was repeated three times with two individual biological isolates. Each time, ∼50 cells were counted for all the samples.

In both cohesion and tethering experiments, confocal microscopy was performed; images were captured using LSM880 (Airyscan) confocal system from Zeiss and were processed and analyzed using ZEN BLACK 2.1 software.

### Telomere accessibility assay

Telomere accessibility assay was performed as before (Kothiwal and Laloraya, 2019). Yeast strains expressing the *E. coli dam* methylase (that methylates adenine within GATC) were grown to log phase and genomic DNA was extracted. Samples were digested overnight at 37°C with DpnI or Sau3AI (methylation sensitive and insensitive restriction endonucleases, respectively, that recognize GATC) and analyzed by quantitative real-time PCR. Primers were designed such that the telomere VIR proximal PCR-fragment generated includes the DpnI/Sau3AI restriction site (∼1.2 kb from the telomere). The fraction of uncut DNA relative to wild-type was measured by qPCR and normalized to an uncut DNA fragment at *ACT1*. Normalization was performed using a primer set (*ACT1-dam*-F/*ACT1-dam*-R) amplifying an uncut sequence at the *ACT1* gene. An unpaired two-tailed Student's *t*-test was used to evaluate the statistical significance of the observed differences in accessibility. (*P≤0.05, **P≤0.01 and ***P≤0.001).

## Supplementary Material

10.1242/joces.265023_sup1 Supplementary information

## References

[JCS265023C1] Adams, A., Gottschling, D. E., Kaiser, C. A. and Stearns, T. (1997). *Methods in yeast genetics: A Cold Spring Harbor Laboratory Course Manual*. New York: Cold Spring Harbor.

[JCS265023C2] Almedawar, S., Colomina, N., Bermudez-Lopez, M., Pocino-Merino, I. and Torres-Rosell, J. (2012). A SUMO-dependent step during establishment of sister chromatid cohesion. *Curr. Biol.* 22, 1576-1581. 10.1016/j.cub.2012.06.046 22771040

[JCS265023C3] Aparicio, O. M., Billington, B. L. and Gottschling, D. E. (1991). Modifiers of position effect are shared between telomeric and silent mating-type loci in *S. cerevisiae*. *Cell* 66, 1279-1287. 10.1016/0092-8674(91)90049-5 1913809

[JCS265023C4] Banerji, R., Skibbens, R. V. and Iovine, M. K. (2017). How many roads lead to cohesinopathies? *Dev. Dyn.* 246, 881-888. 10.1002/dvdy.24510 28422453

[JCS265023C5] Blat, Y. and Kleckner, N. (1999). Cohesins bind to preferential sites along yeast chromosome III, with differential regulation along arms versus the centric region. *Cell* 98, 249-259. 10.1016/S0092-8674(00)81019-3 10428036

[JCS265023C6] Bystricky, K., Laroche, T., van Houwe, G., Blaszczyk, M. and Gasser, S. M. (2005). Chromosome looping in yeast: telomere pairing and coordinated movement reflect anchoring efficiency and territorial organization. *J. Cell Biol.* 168, 375-387. 10.1083/jcb.200409091 15684028 PMC2171726

[JCS265023C7] Deardorff, M. A., Kaur, M., Yaeger, D., Rampuria, A., Korolev, S., Pie, J., Gil-Rodriguez, C., Arnedo, M., Loeys, B., Kline, A. D. et al. (2007). Mutations in cohesin complex members SMC3 and SMC1A cause a mild variant of Cornelia de Lange syndrome with predominant mental retardation. *Am. J. Hum. Genet.* 80, 485-494. 10.1086/511888 17273969 PMC1821101

[JCS265023C8] Donze, D., Adams, C. R., Rine, J. and Kamakaka, R. T. (1999). The boundaries of the silenced *HMR* domain in *Saccharomyces cerevisiae*. *Genes Dev.* 13, 698-708. 10.1101/gad.13.6.698 10090726 PMC316548

[JCS265023C9] Ellahi, A., Thurtle, D. M. and Rine, J. (2015). The chromatin and transcriptional landscape of native *Saccharomyces cerevisiae* telomeres and subtelomeric domains. *Genetics* 200, 505-521. 10.1534/genetics.115.175711 25823445 PMC4492376

[JCS265023C10] Feuerbach, F., Galy, V., Trelles-Sticken, E., Fromont-Racine, M., Jacquier, A., Gilson, E., Olivo-Marin, J. C., Scherthan, H. and Nehrbass, U. (2002). Nuclear architecture and spatial positioning help establish transcriptional states of telomeres in yeast. *Nat. Cell Biol.* 4, 214-221. 10.1038/ncb756 11862215

[JCS265023C11] Gard, S., Light, W., Xiong, B., Bose, T., McNairn, A. J., Harris, B., Fleharty, B., Seidel, C., Brickner, J. H. and Gerton, J. L. (2009). Cohesinopathy mutations disrupt the subnuclear organization of chromatin. *J. Cell Biol.* 187, 455-462. 10.1083/jcb.200906075 19948494 PMC2779225

[JCS265023C12] Gartenberg, M. R. and Smith, J. S. (2016). The nuts and bolts of transcriptionally silent chromatin in *Saccharomyces cerevisiae*. *Genetics* 203, 1563-1599. 10.1534/genetics.112.145243 27516616 PMC4981263

[JCS265023C13] Glynn, E. F., Megee, P. C., Yu, H. G., Mistrot, C., Unal, E., Koshland, D. E., DeRisi, J. L. and Gerton, J. L. (2004). Genome-wide mapping of the cohesin complex in the yeast *Saccharomyces cerevisiae*. *PLoS Biol.* 2, E259. 10.1371/journal.pbio.0020259 15309048 PMC490026

[JCS265023C14] Gottschling, D. E. (1992). Telomere-proximal DNA in *Saccharomyces cerevisiae* is refractory to methyltransferase activity in vivo. *Proc. Natl. Acad. Sci. USA* 89, 4062-4065. 10.1073/pnas.89.9.4062 1570334 PMC525632

[JCS265023C15] Gottschling, D. E., Aparicio, O. M., Billington, B. L. and Zakian, V. A. (1990). Position effect at *S. cerevisiae* telomeres: reversible repression of Pol II transcription. *Cell* 63, 751-762. 10.1016/0092-8674(90)90141-Z 2225075

[JCS265023C16] Guacci, V., Koshland, D. and Strunnikov, A. (1997). A direct link between sister chromatid cohesion and chromosome condensation revealed through the analysis of *MCD1* in *S. cerevisiae*. *Cell* 91, 47-57. 10.1016/S0092-8674(01)80008-8 9335334 PMC2670185

[JCS265023C17] Hardy, C. F., Sussel, L. and Shore, D. (1992). A RAP1-interacting protein involved in transcriptional silencing and telomere length regulation. *Genes Dev.* 6, 801-814. 10.1101/gad.6.5.801 1577274

[JCS265023C18] Hazelrigg, T., Levis, R. and Rubin, G. M. (1984). Transformation of white locus DNA in *Drosophila*: dosage compensation, zeste interaction, and position effects. *Cell* 36, 469-481. 10.1016/0092-8674(84)90240-X 6319027

[JCS265023C19] Hecht, A., Strahl-Bolsinger, S. and Grunstein, M. (1996). Spreading of transcriptional repressor SIR3 from telomeric heterochromatin. *Nature* 383, 92-96. 10.1038/383092a0 8779721

[JCS265023C20] Hediger, F. and Gasser, S. M. (2002). Nuclear organization and silencing: putting things in their place. *Nat. Cell Biol.* 4, E53-E55. 10.1038/ncb0302-e53 11875445

[JCS265023C21] Hornig, N. C. and Uhlmann, F. (2004). Preferential cleavage of chromatin-bound cohesin after targeted phosphorylation by Polo-like kinase. *EMBO J.* 23, 3144-3153. 10.1038/sj.emboj.7600303 15241476 PMC514920

[JCS265023C22] Horsfield, J. A., Print, C. G. and Monnich, M. (2012). Diverse developmental disorders from the one ring: distinct molecular pathways underlie the cohesinopathies. *Front. Genet.* 3, 171. 10.3389/fgene.2012.00171 22988450 PMC3439829

[JCS265023C23] Ivanov, D. and Nasmyth, K. (2005). A topological interaction between cohesin rings and a circular minichromosome. *Cell* 122, 849-860. 10.1016/j.cell.2005.07.018 16179255

[JCS265023C24] Janke, C., Magiera, M. M., Rathfelder, N., Taxis, C., Reber, S., Maekawa, H., Moreno-Borchart, A., Doenges, G., Schwob, E., Schiebel, E. et al. (2004). A versatile toolbox for PCR-based tagging of yeast genes: new fluorescent proteins, more markers and promoter substitution cassettes. *Yeast* 21, 947-962. 10.1002/yea.1142 15334558

[JCS265023C25] Kothiwal, D. and Laloraya, S. (2019). A SIR-independent role for cohesin in subtelomeric silencing and organization. *Proc. Natl. Acad. Sci. USA* 116, 5659-5664. 10.1073/pnas.1816582116 30842278 PMC6431164

[JCS265023C26] Kothiwal, D., Gopinath, S. and Laloraya, S. (2021). Cohesin dysfunction results in cell wall defects in budding yeast. *Genetics* 217, 1-16. 10.1093/genetics/iyaa023 PMC8045705 33683362

[JCS265023C27] Kueng, S., Oppikofer, M. and Gasser, S. M. (2013). SIR proteins and the assembly of silent chromatin in budding yeast. *Annu. Rev. Genet.* 47, 275-306. 10.1146/annurev-genet-021313-173730 24016189

[JCS265023C28] Kyrion, G., Liu, K., Liu, C. and Lustig, A. J. (1993). RAP1 and telomere structure regulate telomere position effects in *Saccharomyces cerevisiae*. *Genes Dev.* 7, 1146-1159. 10.1101/gad.7.7a.1146 8319907

[JCS265023C29] Laloraya, S., Guacci, V. and Koshland, D. (2000). Chromosomal addresses of the cohesin component Mcd1p. *J. Cell Biol.* 151, 1047-1056. 10.1083/jcb.151.5.1047 11086006 PMC2174344

[JCS265023C30] Landry, J., Sutton, A., Tafrov, S. T., Heller, R. C., Stebbins, J., Pillus, L. and Sternglanz, R. (2000). The silencing protein SIR2 and its homologs are NAD-dependent protein deacetylases. *Proc. Natl. Acad. Sci. USA* 97, 5807-5811. 10.1073/pnas.110148297 10811920 PMC18515

[JCS265023C31] Lengronne, A., Katou, Y., Mori, S., Yokobayashi, S., Kelly, G. P., Itoh, T., Watanabe, Y., Shirahige, K. and Uhlmann, F. (2004). Cohesin relocation from sites of chromosomal loading to places of convergent transcription. *Nature* 430, 573-578. 10.1038/nature02742 15229615 PMC2610358

[JCS265023C32] Lindroos, H. B., Strom, L., Itoh, T., Katou, Y., Shirahige, K. and Sjogren, C. (2006). Chromosomal association of the Smc5/6 complex reveals that it functions in differently regulated pathways. *Mol. Cell* 22, 755-767. 10.1016/j.molcel.2006.05.014 16793545

[JCS265023C33] Liu, J., Zhang, Z., Bando, M., Itoh, T., Deardorff, M. A., Clark, D., Kaur, M., Tandy, S., Kondoh, T., Rappaport, E. et al. (2009). Transcriptional dysregulation in NIPBL and cohesin mutant human cells. *PLoS Biol.* 7, e1000119. 10.1371/journal.pbio.1000119 19468298 PMC2680332

[JCS265023C34] McAleenan, A., Cordon-Preciado, V., Clemente-Blanco, A., Liu, I. C., Sen, N., Leonard, J., Jarmuz, A. and Aragon, L. (2012). SUMOylation of the alpha-Kleisin subunit of Cohesin is required for DNA damage-induced cohesion. *Curr. Biol.* 22, 1564-1575. 10.1016/j.cub.2012.06.045 22771042

[JCS265023C35] Michaelis, C., Ciosk, R. and Nasmyth, K. (1997). Cohesins: chromosomal proteins that prevent premature separation of sister chromatids. *Cell* 91, 35-45. 10.1016/S0092-8674(01)80007-6 9335333

[JCS265023C36] Mishra, K. and Shore, D. (1999). Yeast Ku protein plays a direct role in telomeric silencing and counteracts inhibition by rif proteins. *Curr. Biol.* 9, 1123-1126. 10.1016/S0960-9822(99)80483-7 10531008

[JCS265023C37] Moazed, D. (2001). Common themes in mechanisms of gene silencing. *Mol. Cell* 8, 489-498. 10.1016/S1097-2765(01)00340-9 11583612

[JCS265023C38] Neurohr, G., Naegeli, A., Titos, I., Theler, D., Greber, B., Diez, J., Gabaldon, T., Mendoza, M. and Barral, Y. (2011). A midzone-based ruler adjusts chromosome compaction to anaphase spindle length. *Science* 332, 465-468. 10.1126/science.1201578 21393511

[JCS265023C39] Pryde, F. E. and Louis, E. J. (1999). Limitations of silencing at native yeast telomeres. *EMBO J.* 18, 2538-2550. 10.1093/emboj/18.9.2538 10228167 PMC1171335

[JCS265023C40] Renauld, H., Aparicio, O. M., Zierath, P. D., Billington, B. L., Chhablani, S. K. and Gottschling, D. E. (1993). Silent domains are assembled continuously from the telomere and are defined by promoter distance and strength, and by SIR3 dosage. *Genes Dev.* 7, 1133-1145. 10.1101/gad.7.7a.1133 8319906

[JCS265023C41] Rolef Ben-Shahar, T., Heeger, S., Lehane, C., East, P., Flynn, H., Skehel, M. and Uhlmann, F. (2008). Eco1-dependent cohesin acetylation during establishment of sister chromatid cohesion. *Science* 321, 563-566. 10.1126/science.1157774 18653893

[JCS265023C42] Skibbens, R. V., Marzillier, J. and Eastman, L. (2010). Cohesins coordinate gene transcriptions of related function within *Saccharomyces cerevisiae*. *Cell Cycle* 9, 1601-1606. 10.4161/cc.9.8.11307 20404480 PMC3096706

[JCS265023C43] Smith, J. S. and Boeke, J. D. (1997). An unusual form of transcriptional silencing in yeast ribosomal DNA. *Genes Dev.* 11, 241-254. 10.1101/gad.11.2.241 9009206

[JCS265023C44] Stead, K., Aguilar, C., Hartman, T., Drexel, M., Meluh, P. and Guacci, V. (2003). Pds5p regulates the maintenance of sister chromatid cohesion and is sumoylated to promote the dissolution of cohesion. *J. Cell Biol.* 163, 729-741. 10.1083/jcb.200305080 14623866 PMC2173684

[JCS265023C45] Strahl-Bolsinger, S., Hecht, A., Luo, K. and Grunstein, M. (1997). SIR2 and SIR4 interactions differ in core and extended telomeric heterochromatin in yeast. *Genes Dev.* 11, 83-93. 10.1101/gad.11.1.83 9000052

[JCS265023C46] Straight, A. F., Shou, W., Dowd, G. J., Turck, C. W., Deshaies, R. J., Johnson, A. D. and Moazed, D. (1999). Net1, a Sir2-associated nucleolar protein required for rDNA silencing and nucleolar integrity. *Cell* 97, 245-256. 10.1016/S0092-8674(00)80734-5 10219245

[JCS265023C47] Strom, L., Lindroos, H. B., Shirahige, K. and Sjogren, C. (2004). Postreplicative recruitment of cohesin to double-strand breaks is required for DNA repair. *Mol. Cell* 16, 1003-1015. 10.1016/j.molcel.2004.11.026 15610742

[JCS265023C48] Tanaka, T., Cosma, M. P., Wirth, K. and Nasmyth, K. (1999). Identification of cohesin association sites at centromeres and along chromosome arms. *Cell* 98, 847-858. 10.1016/S0092-8674(00)81518-4 10499801

[JCS265023C49] Unal, E., Heidinger-Pauli, J. M., Kim, W., Guacci, V., Onn, I., Gygi, S. P. and Koshland, D. E. (2008). A molecular determinant for the establishment of sister chromatid cohesion. *Science* 321, 566-569. 10.1126/science.1157880 18653894

[JCS265023C50] van der Lelij, P., Chrzanowska, K. H., Godthelp, B. C., Rooimans, M. A., Oostra, A. B., Stumm, M., Zdzienicka, M. Z., Joenje, H. and de Winter, J. P. (2010). Warsaw breakage syndrome, a cohesinopathy associated with mutations in the XPD helicase family member DDX11/ChlR1. *Am. J. Hum. Genet.* 86, 262-266. 10.1016/j.ajhg.2010.01.008 20137776 PMC2820174

[JCS265023C51] Wotton, D. and Shore, D. (1997). A novel Rap1p-interacting factor, Rif2p, cooperates with Rif1p to regulate telomere length in *Saccharomyces cerevisiae*. *Genes Dev.* 11, 748-760. 10.1101/gad.11.6.748 9087429

[JCS265023C52] Wu, N., Kong, X., Ji, Z., Zeng, W., Potts, P. R., Yokomori, K. and Yu, H. (2012). Scc1 sumoylation by Mms21 promotes sister chromatid recombination through counteracting Wapl. *Genes Dev.* 26, 1473-1485. 10.1101/gad.193615.112 22751501 PMC3403015

[JCS265023C53] Wyrick, J. J., Holstege, F. C., Jennings, E. G., Causton, H. C., Shore, D., Grunstein, M., Lander, E. S. and Young, R. A. (1999). Chromosomal landscape of nucleosome-dependent gene expression and silencing in yeast. *Nature* 402, 418-421. 10.1038/46567 10586882

[JCS265023C54] Zhang, J., Shi, X., Li, Y., Kim, B. J., Jia, J., Huang, Z., Yang, T., Fu, X., Jung, S. Y., Wang, Y. et al. (2008). Acetylation of Smc3 by Eco1 is required for S phase sister chromatid cohesion in both human and yeast. *Mol. Cell* 31, 143-151. 10.1016/j.molcel.2008.06.006 18614053

