# Supplementary material for: Cohesin sumoylation is required for repression of subtelomeric gene expression in Saccharomyces cerevisiae
Source: J Cell Sci. 2026 Jun 26;139(12):jcs265023. doi: 10.1242/jcs.265023 (PMC13354957; doi:10.1242/jcs.265023)
Supplement: Supplementary information [file joces-139-265023-s1.pdf]

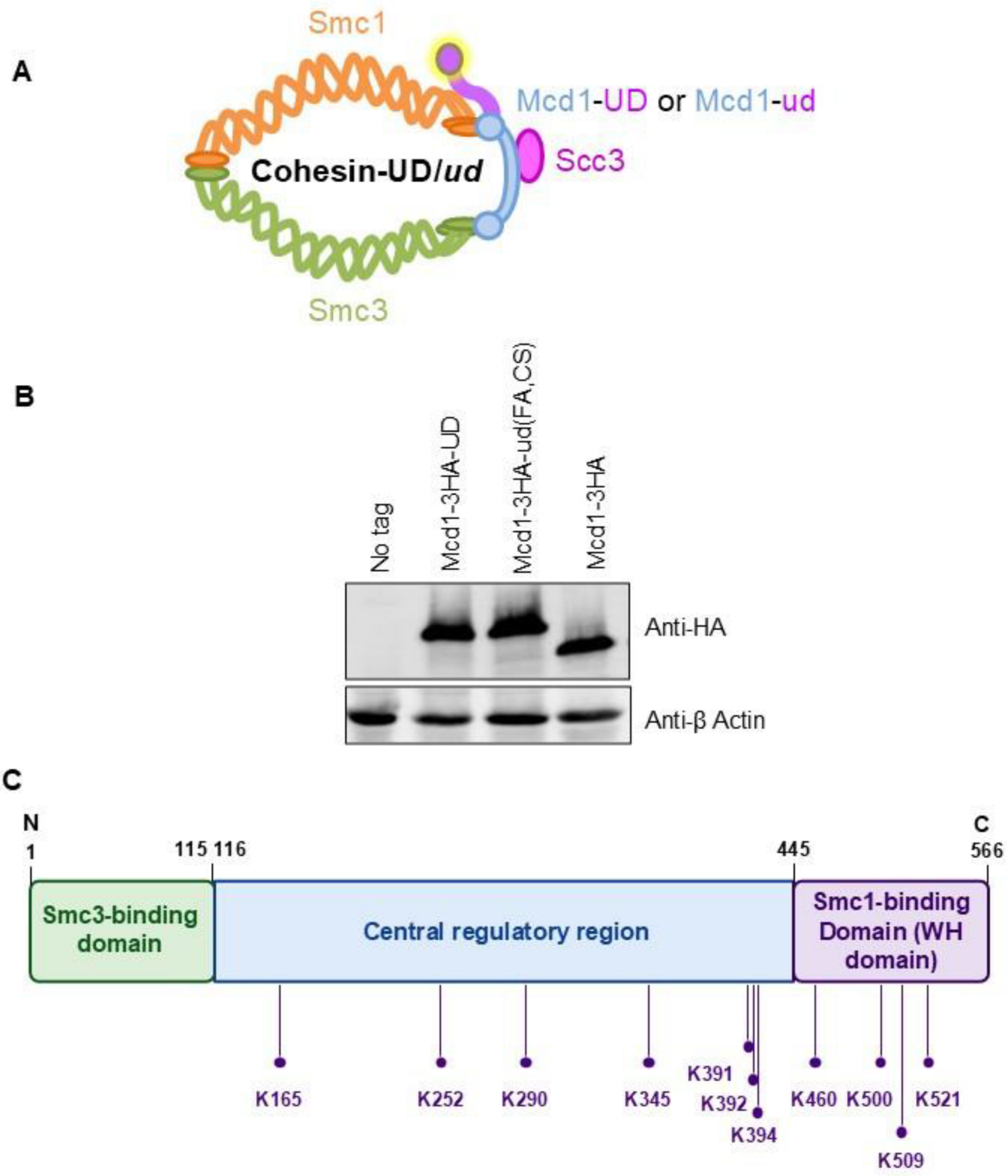

Fig. S1. Details of Mcd1-3HA-UD fusion and *mcd1-SD*. **(A)** Schematic showing fusion of Ulp1 domain, UD (purple) to Mcd1, the kleisin component of the cohesin complex (blue). **(B)** Western blot showing equivalent expression levels of Mcd1-3HA (SLY1807), Mcd1-3HA-UD (SLY1820) and Mcd1-3HA-ud(FA,CS) (SLY1823). β Actin was used as a loading control. (**C**) Schematic showing the locations of the 11 K → R substitutions in *mcd1-SD*.

Journal of Cell Science • Supplementary information

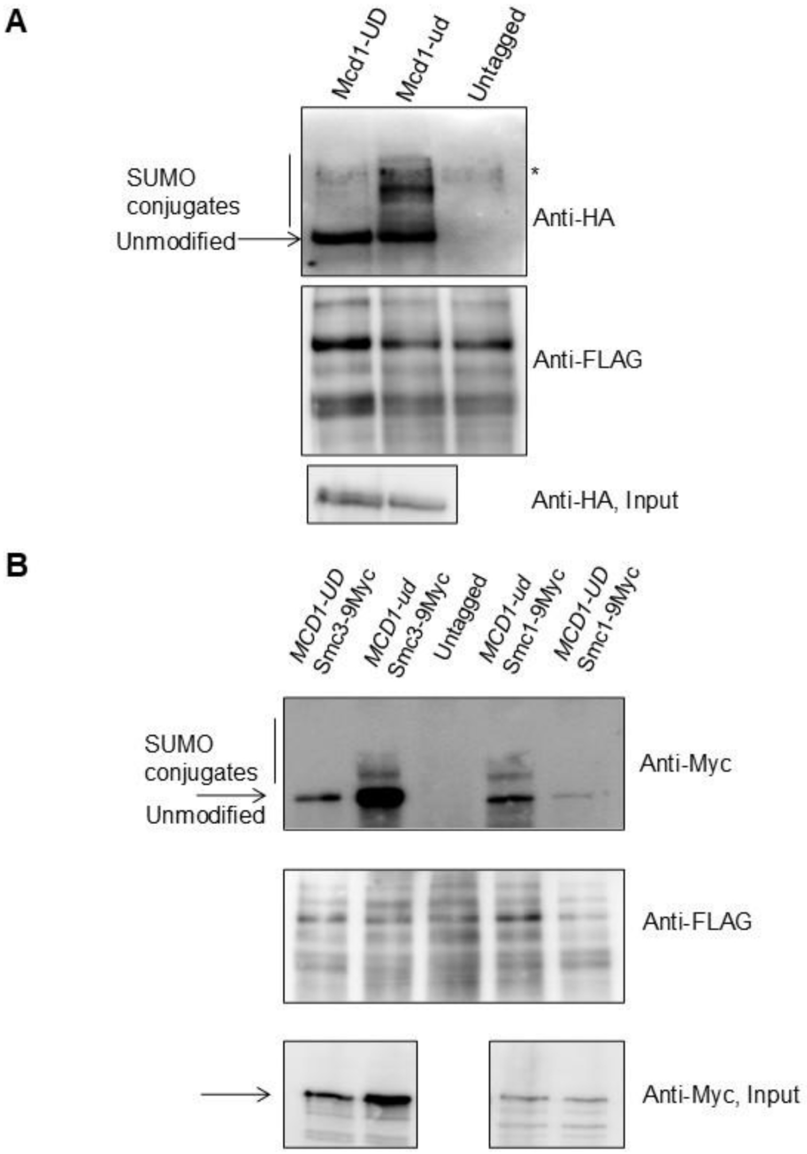

Fig. S2. Fusion of the ULP domain (UD) to Mcd1 can de-sumoylate the cohesin complex. Analysis of sumoylation of different subunits of the cohesin complex. Pull-down assays were carried out using a strain (SLY2480) expressing SUMO with 6His-Flag tag at the N-terminus (HF-SUMO). All the sumoylated proteins in the cell were pulled down using Ni-NTA resin. **(A)** Fusion of active UD (Mcd1-UD) downregulates Mcd1 sumoylation. HF-SUMO strains expressing Mcd1-UD (SLY2905) or Mcd1-ud (SLY2906) were used. After pull-down, the anti-FLAG blot shows the pull-down efficiency, and the anti-HA blot detects the relevant sumoylated HA-tagged protein Mcd1. **(B)** Pull down using Ni-NTA resin and western blotting to detect Smc1-9Myc (SLY2907 and SLY2908) and Smc3-9Myc (SLY2909 and SLY2910) sumoylation in *MCD1-UD* or *MCD1-ud* strains expressing HF-SUMO.

Journal of Cell Science • Supplementary information

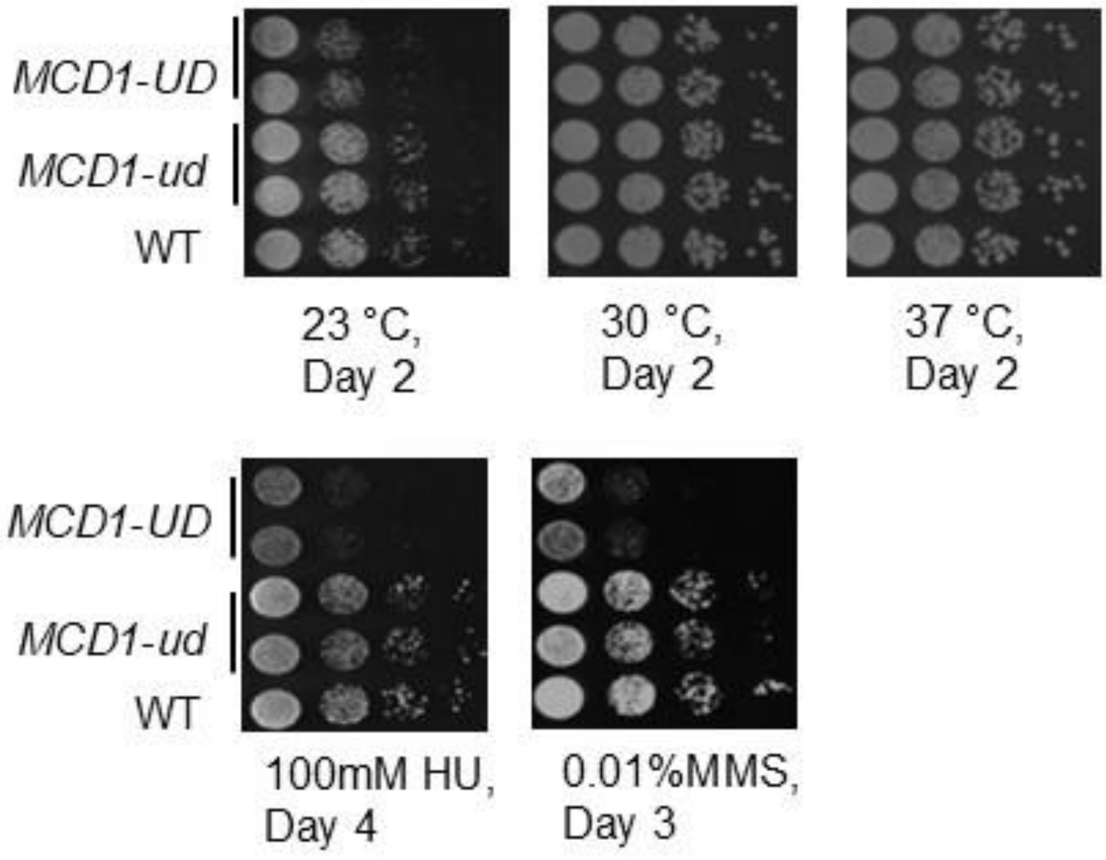

Fig. S3. Characterization of growth defects of *MCD1-UD*. Exponentially growing *MCD1-UD* (SLY1820) or *MCD-ud* (SLY1823) and wild-type (SLY1807)cells were serially diluted (10-fold) and spotted on YPD at the indicated temperatures and YPD containing indicated drugs at 23℃. Images were captured at the indicated times.

Journal of Cell Science • Supplementary information

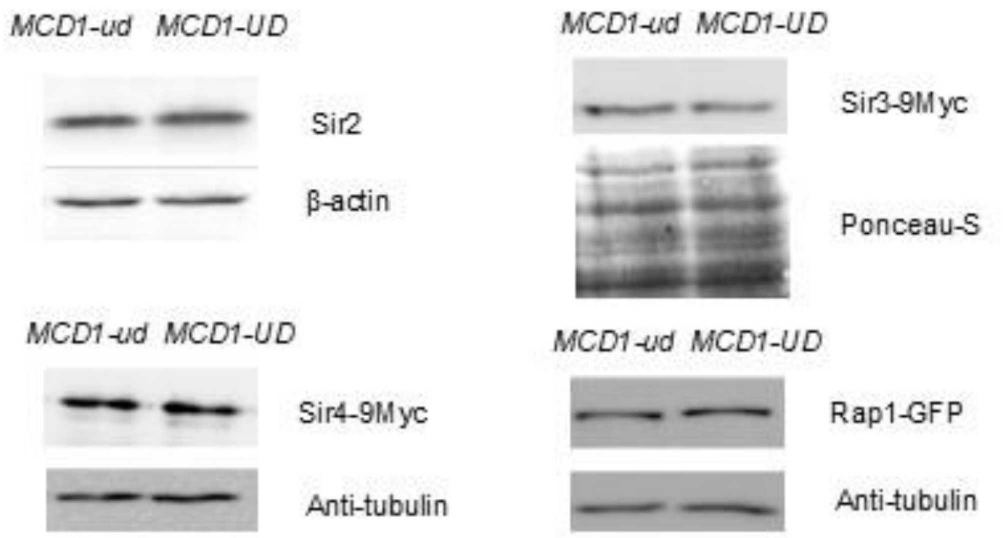

Fig. S4. Steady state level of silencing regulators is not altered in cohesin sumoylation deficient strains. Western blot showing equivalent levels of Sir2 in *MCD1-ud* (SLY1823) and *MCD1-UD* (SLY1820) strains, Sir3-9Myc in *MCD1-ud* (SLY2396) and *MCD1-UD* (SLY2395) strains, Sir4-9Myc (in SLY2387 and SLY2388 that are *MCD1-ud* and *MCD1-UD* respectively) and Rap1-GFP in *MCD1-ud* (SLY2455) and *MCD1-UD* (SLY2457) strains.

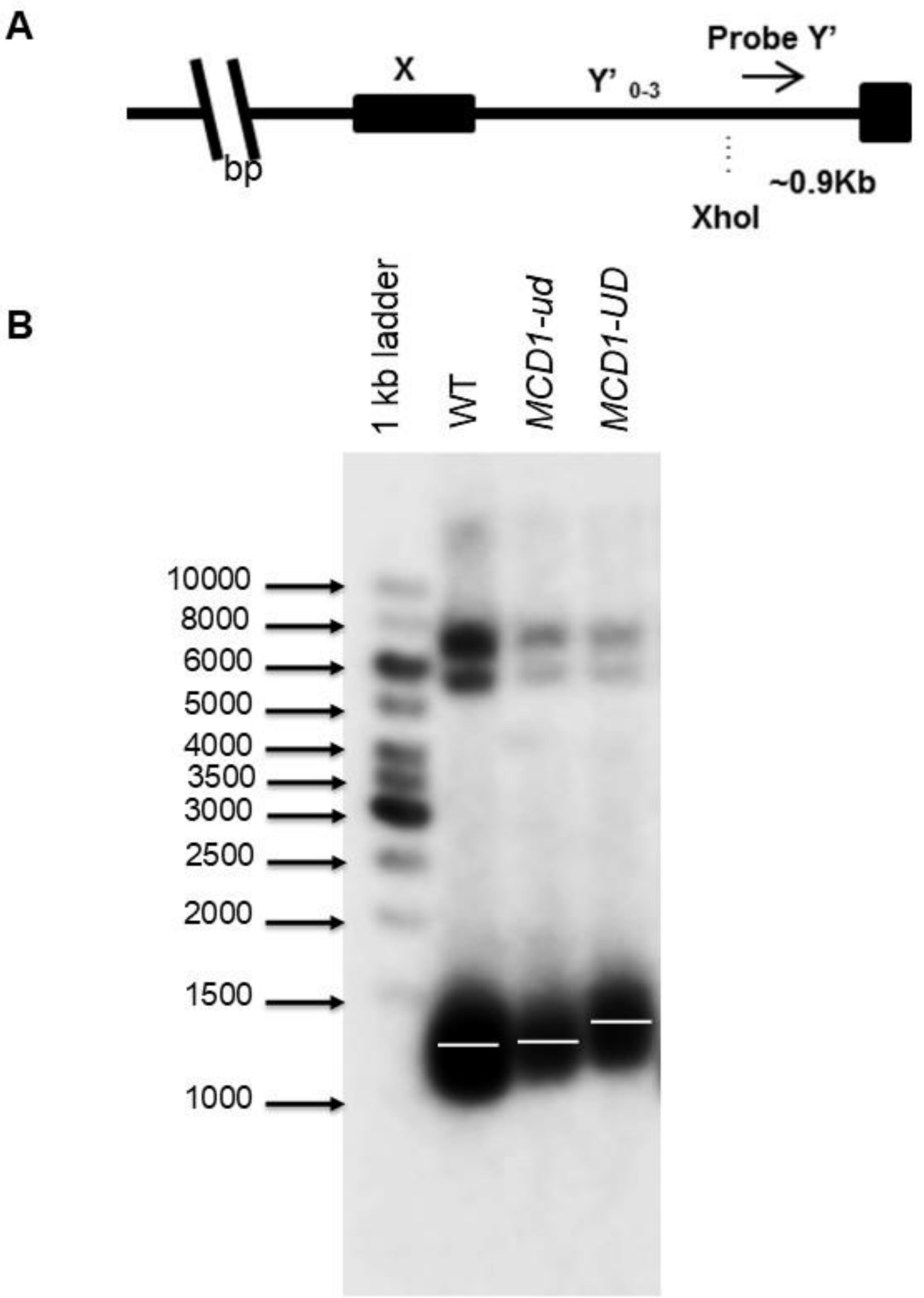

Fig. S5. Telomere length analysis in *MCD1-UD*. **(A)** Schematic depicting a yeast chromosome end, showing the location of the probe fragment relative to the XhoI restriction site and a Y' subtelomeric element. In most yeast telomeres carrying the Y' element, XhoI cuts approximately 950 to 1,300 bp from the junction where the subtelomeric DNA meets the terminal telomeric repeats; when separated on an agarose gel and probed with a Y' specific probe, wild-type Y' telomeres typically appear as a heterogeneous, broad band migrating at around 1.1 to 1.3 kb. **(B)** Southern blot analysis for telomere length measurement in WT (SLY1807), *MCD1-ud* (SLY1823) and *MCD1-UD* (SLY1820). The horizontal white lines indicate the position of the midpoint of the telomere repeat containing Y' Xho1→End fragments for each sample.

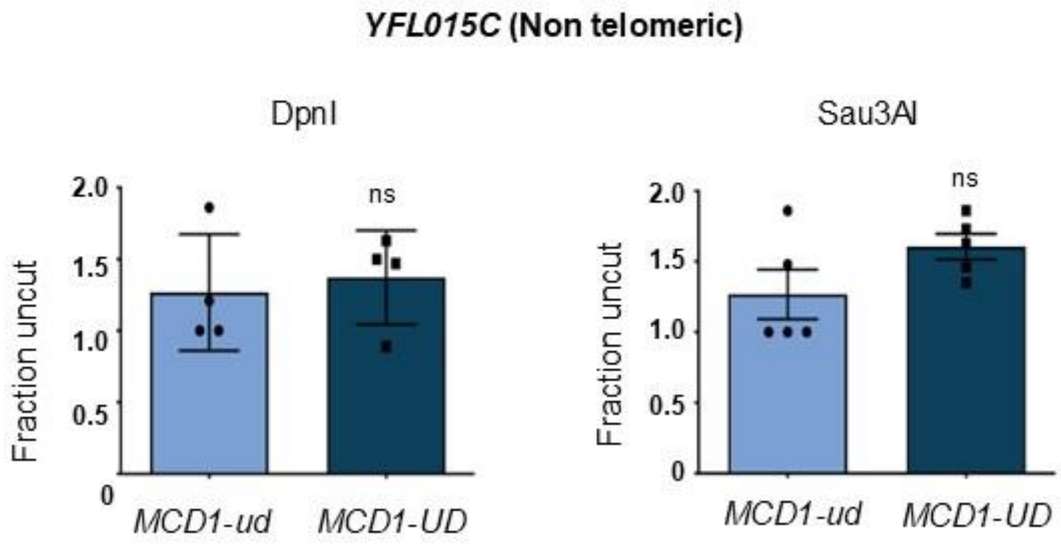

Fig. S6. Assessment of chromatin accessibility at a telomere distal region, *YFL015C*. Dam methylase accessibility assay was carried out using *MCD1-ud* (SLY2996) and *MCD1-UD* (SLY2998) cells by digestion with DpnI (Left) or Sau3AI (Right) followed by qPCR. The mean values for experiments with n=4 independent biological replicates are plotted on the Y-axis, Error bars indicate standard error of the mean (SEM), ns = non-significant.

Journal of Cell Science • Supplementary information

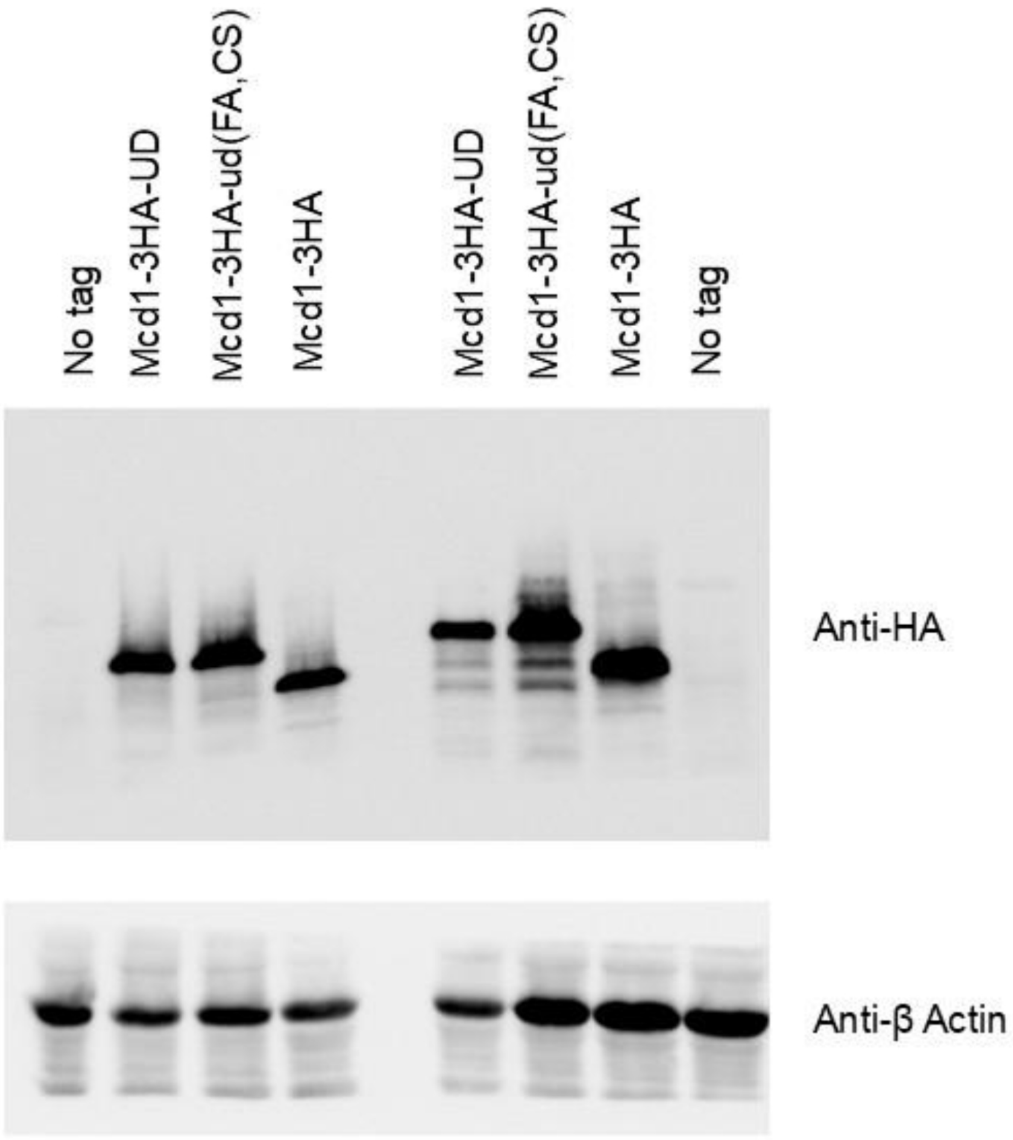

Fig. S7. Uncropped blots from Figure S1B. Top anti-HA to detect Mcd1-3HA and its UD/ud fusion variants, Bottom anti-β Actin. The set of 4 samples on the left are shown in Fig S1B; the set of 4 samples on the right are from a replicate experiment (not included in the Fig S1B).

Journal of Cell Science • Supplementary information

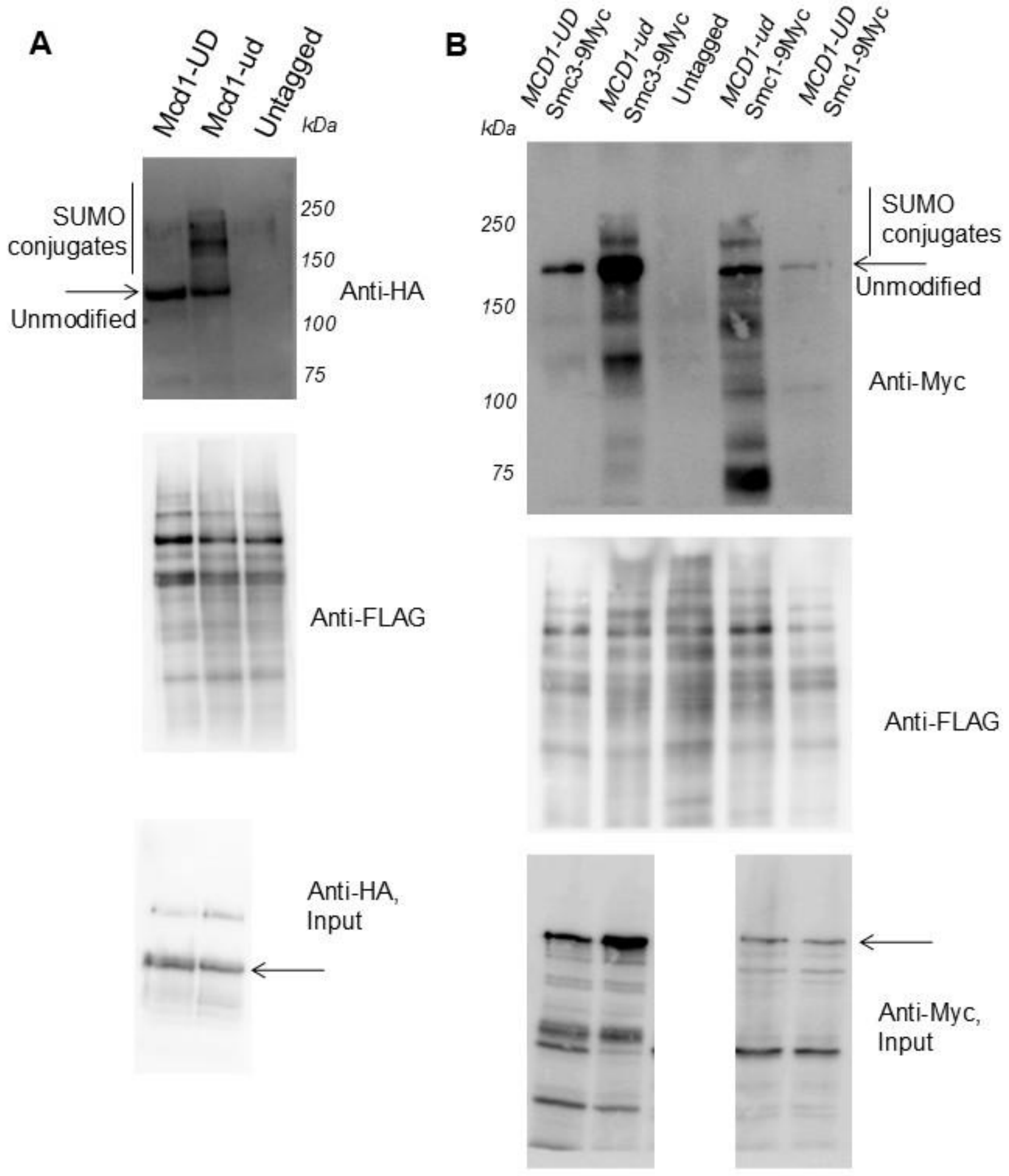

Fig. S8. Uncropped blots from Figure S2. (A) Pull-down of HA-tagged Mcd1-UD or Mcd1-ud with Smt3-FLAG conjugates using Ni-NTA resin. Top anti-HA, Middle anti-FLAG, bottom anti-HA western of input samples. Arrow indicates position of the unmodified Mcd1-3xHA band**. (B)** Pull-down of Myc-tagged Smc3 (left) or Smc1 (Right) from Mcd1-UD or Mcd1-ud expressing strains along with Smt3-FLAG conjugates using Ni-NTA resin. Top anti-Myc, Middle anti-FLAG, bottom anti-Myc western of input samples. Arrow indicates position of the unmodified Smc1/3 -9Myc band.

Journal of Cell Science • Supplementary information

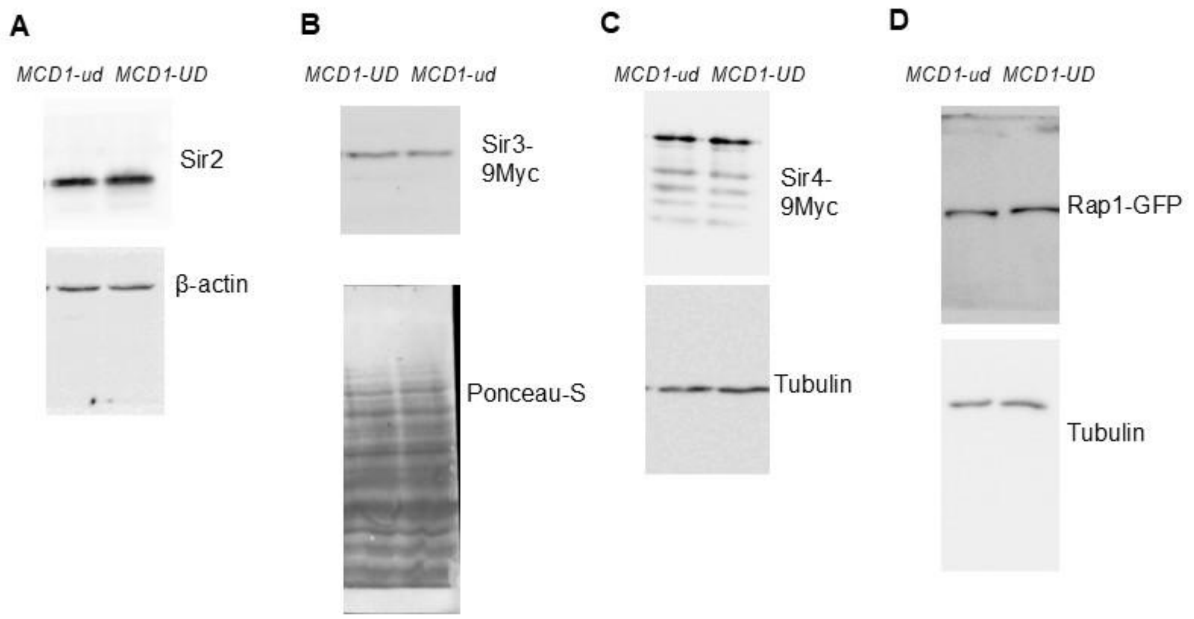

Fig. S9. Uncropped and western blots from Figure S4. (A) anti-Sir2 antibody (top) and anti β-actin (bottom) **(B)** anti-Myc antibody (top) to detect Sir3-9Myc and Ponceau-S stained blot (bottom) **(C)** anti-Myc antibody (top) to detect Sir4-9Myc and anti-tubulin antibody (bottom) **(D)** anti-GFP antibody (top) to detect Rap1-GFP and anti-tubulin antibody (bottom), replicate.

Journal of Cell Science • Supplementary information

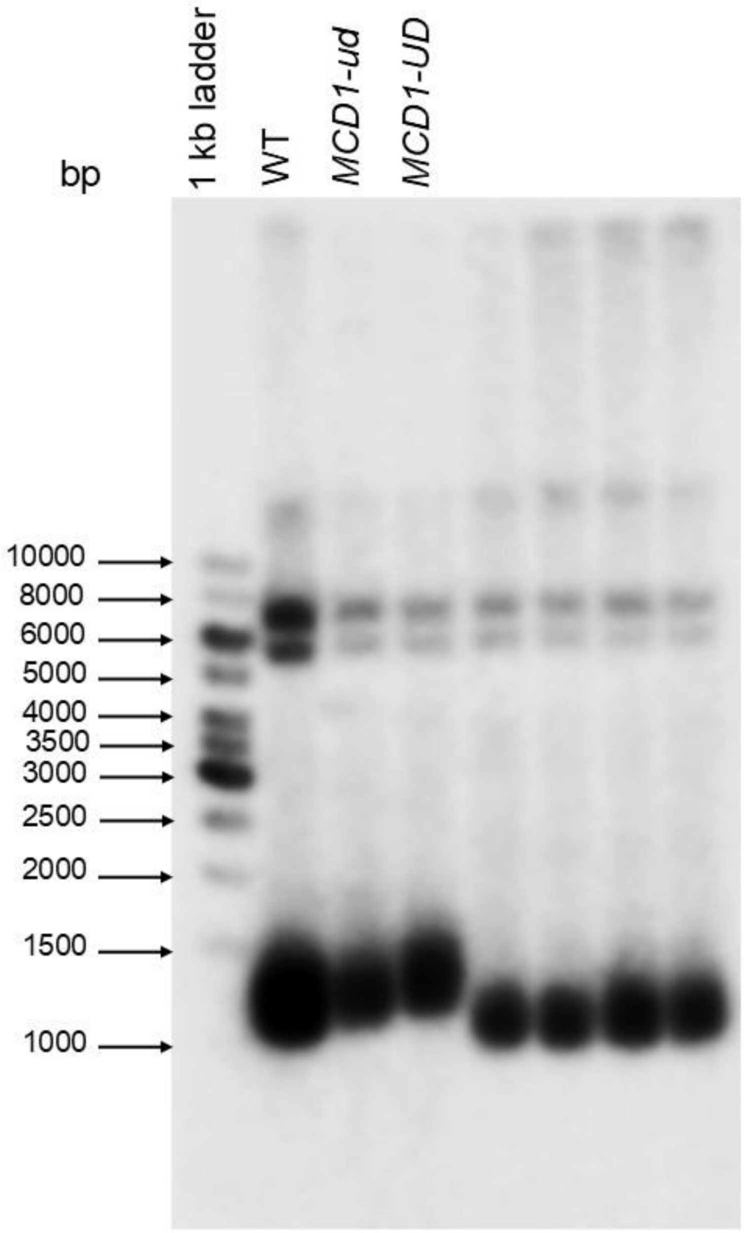

Fig. S10. Uncropped Southern blot from Fig. S5.

Journal of Cell Science • Supplementary information

Table S1. Yeast Strains used in this study

Strain	Genotype	Source/Ref.
ROY 783	*MATa ADE2 leu2-3,112 his3-11,15 ura3-1 trp1-1 ppr1D::HIS3 URA3-TEL-VR*	Donze D 1999 G&D
SLY1820	ROY 783 *MCD1-3HA-UD:LEU2*	This study
SLY1823	ROY 783 *MCD1-3HA-ud:LEU2*	This study
SLY1807	ROY783 *MCD1-3HA:TRP1*	This study
SLY2480	*MATa ade2-1 ura3-1 his3-11,15 trp1-1 leu2-3,112 can1-100 smt3Δ::hphNT1,* pYRTAG310	Lakshmi Mahendrawada
SLY2905	SLY2480 *MCD1-3HA-UD:LEU2*	This study
SLY2906	SLY2480 *MCD1-3HA-ud:LEU2*	This study
SLY2907	SLY2905 *SMC1-9Myc:natNT2*	This study
SLY2908	SLY2906 *SMC1-9Myc:natNT2*	This study
SLY2909	SLY2905 *SMC3-9Myc:natNT2*	This study
SLY2910	SLY2906 *SMC3-9Myc:natNT2*	This study
SLY2743	ROY783 *MCD1-6HA:hphNT1 KanMX6*	This study
SLY2745	ROY783 *mcd1 SD-6HA:hphNT1 KanMX6*	This study
SLY2387	SLY1823 *SIR4-9Myc:hphNT1*	This study
SLY2388	SLY1820 *SIR4-9Myc:hphNT1*	This study
SLY2395	SLY1820 *SIR3-9Myc:hphNT1*	This study
SLY2396	SLY1823 *SIR3-9Myc:hphNT1*	This study
SLY2455	W303-1a *MCD1-ud:LEU2 RAP1-GFP:hphNT1*	This study
SLY2457	W303-1a *MCD1-UD:LEU2 RAP1-GFP:hphNT1*	This study
SLY2526	SLY1823 *rif1Δ::KanMX4*	This study
SLY2528	SLY1820 *rif1Δ::KanMX4*	This study
SLY2911	SLY1823 *sir2Δ::TRP1*	This study
SLY2912	SLY1820 *sir2Δ::TRP1*	This study
GA2201	*MATa ade2-1 can1-100 his3-11,-15 leu2-3,-112 trp1-1 ura3-1 ade2-1::HIS3p-CFP-lacI-URA3p-tetR-YFP-ADE2 TELVI-L::tetO-LEU2, TELVI-R::lacO-TRP1*	Kerstin Bystricky 2005 JCB

Journal of Cell Science • Supplementary information

SLY2446	GA2201 *Mcd1-3HA-ud:KanMX4*	This study
SLY2448	GA2201 *Mcd1-3HA-UD:KanMX4*	This study
yYB3476	*MATa ura3-52 his3Δ200 leu2 lys2-801 ade2-101 trp1Δ63 trp1::TetO:TRP1 lys4::LacO:LEU2 his3::LacR-GFP:HIS3 TetR-mRFP*	Neurohr G 2011 Science
SLY2549	yYB3476 *Mcd1-3HA-ud:KanMX4*	This study
SLY2551	yYB3476 *Mcd1-3HA-UD:KanMX4*	This study
GA2198	*MATa ade2-1 can1-100 his3-11,-15 leu2-3,-112 trp1-1 ura3-1 his3-11,-15::HISp-GFP-LacI-HIS3, nup49::NUP49-GFP TELV-R::lacO:TRP1*	Kerstin Bystricky 2005 JCB
SLY2557	GA2198 *Mcd1-3HA-ud:LEU2*	This study
SLY2559	GA2198 *Mcd1-3HA-UD:LEU2*	This study
SLY2996	*MATa leu2-3,112 trp1-1 can1-100 ura3-1 ade2-1 his3-11,15 Mcd1-3HA-ud:LEU2 dam$^+$ hphNT1*	This study
SLY2998	*MATa leu2-3,112 trp1-1 can1-100 ura3-1 ade2-1 his3-11,15 Mcd1-3HA-UD:LEU2 dam$^+$ hphNT1*	This study

Table S2. Oligonucleotides used in this study

SLO-024	TCTATACTGATACCACGCCT
SLO-441	CGCTATACTGCTGTCGATTC
SLO-534	GTATCTAGCAGCAGAACCGG
SLO-603	GAAATATTAAAATAGACGCCAAACCTGCACTATTTGAAAGGTTTATC AATGCTCGTACGCTGCAGGTCGAC
SLO-604	GAAATATTAAAATAGACGCCAAACCTGCACTATTTGAAAGGTTTATC AATGCTCGTACGCTGCAGGTCGAC
SLO-613	CTCTGGCTGCTAATGTACC
SLO-679	CTTCGGTAGACACATTCAAACCATTTTTCCCTCATCGGCACATTAAA GCTGGATGCGTACGCTGCAGGTCGAC
SLO-680	TATGTAAATTGATATTAATTTGGCACTTTTAAATTATTAAATTGCCTTC TACTTAATCGATGAATTCGAGCTCG
SLO-791	CGAGCTCGAATTCATCGAT
SLO-816	GGACAGAAGGACCCAGTTCAGTTCTAGTTTTACAAATAAATACACGA GCGCGTACGCTGCAGGTCGAC
SLO-817	TGGGGGGAAGGGAGAGGTTTGTTGGCGTTTTTTAGGCATTGTTAAG AGTCATCGATGAATTCGAGCTCG
SLO-818	GAGTTGCCGTGCCTTTCC
SLO-871	ATTAACAAATTGATGGAAAAAGATTTTCAAGTGAATAAGGAGATAAA ACCGTATCGTACGCTGCAGGTCGAC
SLO-872	CAAAGAAAAACAGGGTACACTTCGTTACTGGTCTTTTGTAGAATGAT AAAAAGTCAATCGATGAATTCGAGCTCG
SLO-876	CTGCATGTGTACATAGGCATATCTATGGCGGAAGTGAAAATGAATGT TGGTGGTCAATCGATGAATTCGAGCTCG
SLO-877	GAAATAAATTACGCCTTTTCGATGGATGAAGAATTCAAAA ATATGGACTGCATTCGTACGCTGCAGGTCGAC
SLO-882	GCATTATAATCGCGAAAAAG
SLO-883	ATTATCCTAATTCCCAACTG
SLO-884	CGGTAGCATTTCCATCATA
SLO-907	GGAACTGGTAGAATGGAAATGAGGAAAAGATTTTTTGAGAAGGACC TGTTACGTACGCTGCAGGTCGAC

SLO-908	AAATAAAGGAGTAAAATAAGTTAAACAATGATGTTACTTAATTCAATT ACTCAATCGATGAATTCGAGCTCG
SL-LEU-L2B	CCTTGCGTTTCAGCTTCCACTA

Table S3. qPCR primers used in this study

Primer	Sequence	
SLO-853	CTAGTGTCTATAGTAAGTGCTCGG	*YFR057w* FP
SLO-854	GGTATATTGCCACGCAAAGAAAGG	*YFR057w* RP
SLO-867	GGAAACGTAGAAGGCTGGAACGTT	*ACT1* FP
SLO-868	ACAACGAATTGAGAGTTGCCCCAG	*ACT1* RP
SLO-859	GAATCGATACAACCTTGGCACTC	*PHO5* FP
SLO-860	GGTAATCTCGAATTTGCTTGCTC	*PHO5* RP
SLO-1025	GCTGAGATAAGTAATATCGTTGATGAA	TEL6R-*dam*-F
SLO-1026	TCAAACAAGTAGGAATGCGAAAG	TEL6R-*dam*-R
SLO-1027	AGGTTGCTGCTTTGGTTA	*ACT1-dam*-F
SLO-1028	CGTAGGAGTCTTTTTGACCC	*ACT1-dam*-R
SLO-1080	TCAACACGTCGTCATTGC	NT-*dam*-FP
SLO-1081	CGCAAATCCAAGTGAAAATC	NT-*dam*-RP

Journal of Cell Science • Supplementary information
